# Branched oligosaccharides cause atypical starch granule initiation in Arabidopsis chloroplasts

**DOI:** 10.1093/plphys/kiaf002

**Published:** 2025-01-09

**Authors:** Arvid J M Heutinck, Selina Camenisch, Michaela Fischer-Stettler, Mayank Sharma, Barbara Pfister, Simona Eicke, Chun Liu, Samuel C Zeeman

**Affiliations:** Institute of Molecular Plant Biology, Department of Biology, ETH Zurich, 8092 Zurich, Switzerland; Institute of Molecular Plant Biology, Department of Biology, ETH Zurich, 8092 Zurich, Switzerland; Institute of Molecular Plant Biology, Department of Biology, ETH Zurich, 8092 Zurich, Switzerland; Institute of Molecular Plant Biology, Department of Biology, ETH Zurich, 8092 Zurich, Switzerland; Institute of Molecular Plant Biology, Department of Biology, ETH Zurich, 8092 Zurich, Switzerland; Institute of Molecular Plant Biology, Department of Biology, ETH Zurich, 8092 Zurich, Switzerland; Institute of Molecular Plant Biology, Department of Biology, ETH Zurich, 8092 Zurich, Switzerland; Institute of Molecular Plant Biology, Department of Biology, ETH Zurich, 8092 Zurich, Switzerland

## Abstract

Plant chloroplasts store starch during the day, which acts as a source of carbohydrates and energy at night. Starch granule initiation relies on the elongation of malto-oligosaccharide primers. In *Arabidopsis thaliana*, PROTEIN TARGETING TO STARCH 2 (PTST2) and STARCH SYNTHASE 4 (SS4) are essential for the selective binding and elongation of malto-oligosaccharide primers, respectively, and very few granules are initiated in their absence. However, the precise origin and metabolism of the primers remain unknown. Potential origins of malto-oligosaccharide primers include de novo biosynthesis or their release from existing starch granules. For example, the endoamylase α-AMYLASE 3 (AMY3) can cleave a range of malto-oligosaccharides from the granule surface during starch degradation at night, some of which are branched. In the Arabidopsis double mutant deficient in the two debranching enzymes ISOAMYLASE 3 (ISA3) and LIMIT DEXTRINASE (LDA), branched malto-oligosaccharides accumulate in the chloroplast stroma. Here, we reveal that the *isa3 lda* double mutant shows a substantial increase in granule number per chloroplast, caused by these branched malto-oligosaccharides. The *amy3 isa3 lda* triple mutant, which lacks branched malto-oligosaccharides, has far fewer granules than *isa3 lda*, and its granule numbers are barely higher than in the wild type. Plants lacking both ISA3 and LDA and either PTST2 or SS4 show granule over-initiation, indicating that this process occurs independently of the recently described granule initiation pathway. Our findings provide insight into how and where starch granules are initiated. This knowledge can be used to alter granule number and morphological characteristics, traits known to affect starch properties.

## Teaser

When starch degradation in chloroplasts at night is defective, such that soluble branched malto-oligosaccharides accumulate, these in turn cause excess starch granule initiations during the day.

## Introduction

Starch is the major storage carbohydrate in plants, produced in plastids—either the chloroplasts of leaves (transitory starch) or the amyloplasts of heterotrophic organs (storage starch). It is made up of two glucose polymers: amylose and amylopectin. In both polymers most glucose residues are linked through α-1,4-glycosidic bonds, forming linear chains. These chains are connected at branch points with α-1,6-glycosidic linkages. Amylose has very few branch points, whereas around 4% to 6% of linkages in amylopectin are branching points (Pérez and Bertoft 2010). The branch points in amylopectin are clustered, allowing an ordered packing of double helices formed by unbranched chain segments—a process recently proposed to be protein-mediated (Liu et al. 2023). This results in the formation of crystalline lamellae that alternate with amorphous lamellae containing the branch points (Nakamura and Kainuma 2022). The resultant starch granules are insoluble and relatively inert.

Several classes of enzymes are involved in producing the polymers that comprise starch. The synthesis of amylopectin depends on starch synthases (SSs, which transfer glucose units from ADP-glucose onto the nonreducing ends of glucan chains) and branching enzymes (BEs, which produce branch points by glucan transfer between existing chains, cutting an α-1,4-bond and creating an α-1,6-linkage) (Tetlow and Emes 2014; Pfister and Zeeman 2016). A specialized debranching enzyme (DBE), typically containing ISOAMYLASE 1 and ISOAMYLASE 2 (ISA1 and ISA2) subunits, hydrolyses misplaced branches, promoting the formation of a crystallization-competent amylopectin structure (Ball et al. 1996; Streb et al. 2009).

In leaf chloroplasts, photo-assimilated carbon is partitioned into starch to ensure that there is a supply of sugars at night. In rapidly growing *Arabidopsis thaliana* (Arabidopsis), this transitory starch is almost fully depleted during the night (Smith and Zeeman 2020). Interestingly, this does not eliminate as many granules as might be expected. For example, Bürgy et al. (2021) recently showed that while 97% of transitory starch in Arabidopsis leaves is degraded during the night, the cores of 35% of the granules persisted. Fully degraded granules are replaced by newly initiated ones via a controlled process that creates a balance between chloroplast size and granule number (Crumpton-Taylor et al. 2012). Granule initiation determines not only the number of granules in a plastid, it also affects their shape, size, and simple versus compound nature (Abt and Zeeman 2020; Hawkins et al. 2021; Watson-Lazowski et al. 2022).

There is no convincing evidence that plants have a glucan-priming protein analogous to the self-glucosylating glycogenin found in fungi and animals (D’Hulst and Mérida 2010). Rather, granule initiation most likely requires malto-oligosaccharide (MOS) primers. These primers must be extended, branched, and elaborated before becoming a granule initial. In recent years, much has been discovered about the proteins involved in this process (Seung and Smith 2019; Abt and Zeeman 2020; Merida and Fettke 2021). The principal catalytically active protein in granule initiation is STARCH SYNTHASE 4 (SS4; Roldán et al. 2007). In its absence, chloroplasts contain fewer, aberrantly shaped starch granules, and often lack starch altogether. This phenotype is most severe in the young newly emerging leaves. SS4 is able to elongate soluble MOS and is likely provided with substrates via its physical interaction with PROTEIN TARGETING TO STARCH 2 (PTST2). PTST2 has a carbohydrate-binding module and is proposed to capture linear oligosaccharides with a degree of polymerization of 10 or more—long enough to adopt helical conformations (Seung et al. 2017). In the absence of PTST2, chloroplasts again contain fewer starch granules, while its overexpression causes over-initiation. SS4 and PTST2 are part of a larger group of proteins involved in granule initiation, including MAR-BINDING FILAMENT PROTEIN 1 (MFP1), MYOSIN-RESEMBLING CHLOROPLAST PROTEIN (MRC/PII1; Seung et al. 2018; Vandromme et al. 2019), and SS5, a nonenzymatic homolog of SS4 (Abt et al. 2020). MFP1 is thylakoid-associated and interacts with PTST2, influencing its localization into sub-chloroplastic puncta. It is proposed that starch biosynthetic enzymes and oligosaccharide substrates may be concentrated in these regions, thereby promoting the formation of semi-crystalline granule initials (Seung and Smith 2019; Sharma et al. 2024).

Although much has recently been discovered about the proteins involved in granule initiation, the precise origin and metabolism of the priming glucans, malto-oligosaccharides, remain unknown. MOS can be generated de novo in absence of starch, for example via the actions of α-glucan phosphorylases and glucosyl-/glucano-transferases (Linden et al. 1975; Merida and Fettke 2021). However, most are likely generated as by-products of starch synthesis (i.e. through the action of the trimming isoamylase) or as intermediates of starch breakdown.

Starch breakdown at night has been studied in detail in Arabidopsis leaves, where a number of enzymes collectively attack the starch granule surface and metabolize soluble MOS in the chloroplast stroma. First, the crystalline lamellae of amylopectin are disrupted via glucan phosphorylation. Initially, the C6 positions of a small number of glucosyl residues are phosphorylated by GLUCAN, WATER DIKINASE (Hejazi et al. 2008). Subsequently, the C3 positions of other glucosyl residues nearby are phosphorylated by PHOSPHOGLUCAN, WATER DIKINASE (Hejazi et al. 2009). These phosphorylation events are thought to solubilize the granule surface, rendering it more susceptible to amylolytic degradation.

The most active starch breakdown enzymes are β-amylases. These exo-amylases cleave the penultimate α-1,4-bonds at the nonreducing ends of the chains, producing maltose. β-Amylases cannot break down starch entirely, because they cannot cleave chains with a phosphate group near the nonreducing end, nor act close to an α-1,6-branch point (Takeda and Hizukuri 1981). Other enzymes are needed to remove these obstacles: phosphate groups are removed by the phosphoglucan phosphatases STARCH EXCESS 4 (SEX4) and LIKE SEX4 2 (LSF2; Santelia et al. 2011; Kötting et al. 2009). The short stubs of branches are cleaved off by two DBEs, ISOAMYLASE 3 (ISA3) and LIMIT DEXTRINASE (LDA), which hydrolyze the α-1,6 branch points (Wattebled et al. 2005; Delatte et al. 2006). Furthermore, the chloroplastic α-AMYLASE 3 (AMY3) can act endo-amylolytically, cleaving internal α-1,4-bonds and liberating a mixture of linear, branched, and even phosphorylated MOS (Delatte et al. 2006; Kötting et al. 2009).

Blocking key steps in starch breakdown reduces the rate of degradation, leading to an imbalance between daytime starch synthesis and nighttime degradation and consequently excess starch accumulation in leaves. This is the case when ISA3 or both ISA3 and LDA are missing; nocturnal starch breakdown is incomplete and, in the double mutant, soluble MOS accumulate during the night to ∼10 times the level seen in the wild type (Delatte et al. 2006). These MOS are branched and are liberated from the granule by AMY3, which is induced in the *isa3 lda* double mutant (Wattebled et al. 2005; Delatte et al. 2006; Streb et al. 2012). The ISA1/ISA2 DBE is evidently unable to compensate for the loss of ISA3 and LDA, presumably because it preferentially hydrolyzes branch points of longer internal chains during starch synthesis rather than short external stubs created by β-amylase during degradation (Streb et al. 2012).

Here, we used the *isa3 lda* double mutant to study the impact of altered MOS levels on starch granule initiation events. Our data show that branched oligosaccharides can serve as primers and cause atypical granule initiation independently of the key granule initiating proteins SS4 and PTST2.

## Results

### *The isa3 lda* double mutant shows drastically increased granule number in mature leaves

**Figure 1.**
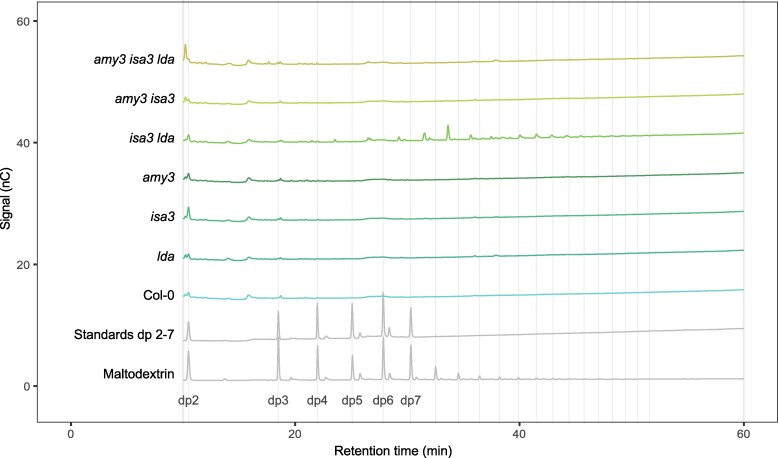
Malto-oligosaccharides of plants deficient in starch breakdown enzymes. Whole rosettes were harvested at the end of the dark period. Malto-oligosaccharides were extracted and analyzed using HPAEC-PAD. Equal amounts of fresh weight equivalent (2.5 mg) were loaded per sample. Debranched maltodextrin was used to determine elution times of linear glucans, the first peak shown corresponds to maltose (this chromatogram shown is scaled down to fit the plot). Branched oligosaccharides elute slightly earlier, causing interlinear peaks. Results were consistent across biological replicates (*n* = 4 plants), representative chromatograms are shown. See also Supplementary Fig. S2. The dp 2 to 7 standards have a concentration of 1 μM.

First, we reconfirmed the presence of branched MOS during starch breakdown in the *isa3 lda* double mutant (Delatte et al. 2006; Streb et al. 2012). Soluble sugars were extracted from whole rosettes harvested at the end of the night and analyzed using high-performance anion-exchange chromatography with pulsed amperometric detection (HPAEC-PAD). Branched oligosaccharides elute slightly earlier than linear glucans with the same degree of polymerization (dp) and disappear when treating the sample with debranching enzymes (Delatte et al. 2006). The chromatograms revealed the presence of branched MOS in *isa3 lda*, but not in the wild type or the *isa3* and *lda* single mutants (Fig. 1; Supplementary Figs. S1 and S2). The branched MOS had a dp between 8 and 20, consistent with earlier reports (Fig. 1). There were no detectable amounts of linear MOS (except for maltose) in the soluble fraction. The amount and size distribution of the branched MOS were similar between extracts prepared separately from *isa3 lda* rosettes split into younger and more mature leaves (Supplementary Fig. S3).

As previously reported (Delatte et al. 2006; Streb et al. 2012) mutants lacking ISA3 accumulate starch due to incomplete starch breakdown overnight (Supplementary Fig. S4). At the end of night, mature leaves of wild-type and *lda* plants contained almost no starch (0.2 ± 0.2 and 1.0 ± 0.9 mg/g fresh weight, respectively), whereas mature leaves of *isa3* and *isa3 lda* contained increasing amounts of starch (12.9 ± 3.3 and 23.2 ± 2.4 mg/g fresh weight, respectively). Young leaves of *isa3* and *isa3 lda* had less starch than mature leaves. Lugol's staining confirmed that the starch-excess phenotype developed with leaf age (Supplementary Fig. S5).

We analyzed the activities of a range of other starch metabolic enzymes using semiquantitative zymograms and assayed DISPROPORTIONATING ENZYME (DPE1) using an enzyme-linked assay. All of the monitored activities were present in all of the lines (except where respective mutant lines were used as controls). We did not observe consistent differences in the activities of plastidial ALPHA-GLUCAN PHOSPHORYLASE 1 (PHS1) or the cytosolic, maltose-specific DISPROPORTIONATING ENZYME 2 (DPE2; Supplementary Fig. S6). Several lines, including *isa3* and *isa3 lda*, had apparent increases in the activities of cytosolic α-GLUCAN PHOSPHORYLASE 2. The plastidial DPE1 was unchanged in most lines but slightly elevated in *isa3*. There were apparent decreases in the activities of STARCH SYNTHASE 1, STARCH SYNTHASE 3 (SS3), BRANCHING ENZYME 2 (BE2), and BRANCHING ENZYME 3 (BE3) in *isa3 lda* and other higher order mutants (Supplementary Fig. S6).

**Figure 2.**
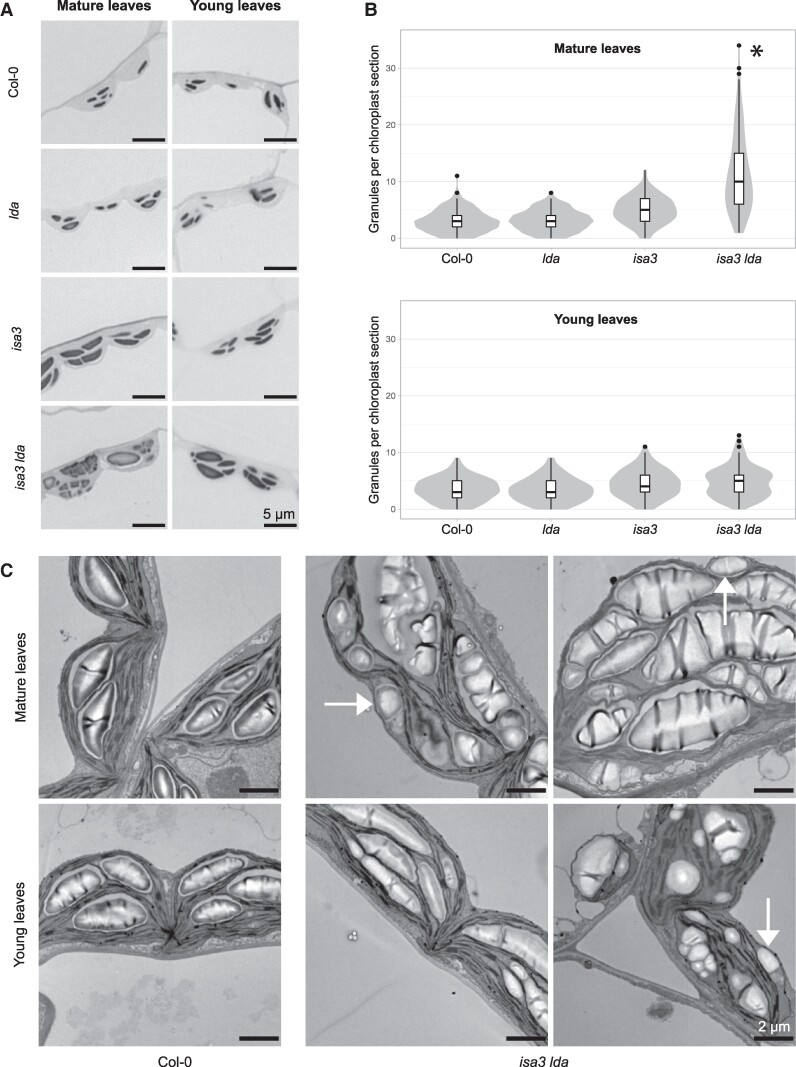
Starch granule phenotypes in the absence of ISA3 and LDA. **A)** Chloroplasts imaged using LM. Leaf samples were harvested at the end of the light period and stained with Toluidine Blue. Representative images are shown. **B)** Distribution of the amount of granules visible per chloroplast section in light micrographs. Box plots show median granule number (center line), box limits correspond to the first- and third-quartile. Whiskers extend up to 1.5 times the inter-quartile range from the box limits, points beyond that are plotted individually as outliers. Granule number was determined for 100 chloroplasts for each of 3 biological replicate plants per genotype. Statistics were done on the biological replicate level using ANOVA followed by Tukey's Honestly Significant Difference test (*P_adj_ < 0.05). **C)** Transmission electron micrographs of Col-0 and *isa3 lda* harvested at the end of the light period. Arrows indicate granules located at the envelope membrane.

Next, light microscopy (LM) was used to examine large numbers of palisade mesophyll cell chloroplasts in replicate sections of mature leaves (Fig. 2A; Supplementary Fig. S7). Starch granules visible in each chloroplast section were counted (Fig. 2B). Because these are thin 2D sections, the number of granules per chloroplast section does not necessarily represent the total number within the chloroplast. Furthermore, starch granule size will influence the likelihood of being captured within the plane of a section. Wild-type and *lda* plants, which had similar starch contents (Delatte et al. 2006; Supplementary Fig. S4), contained 3.0 ± 0.9 and 3.1 ± 0.5 granules per chloroplast section, respectively. The *isa3* mutant, which has a starch-excess phenotype (Delatte et al. 2006; Wattebled et al. 2005; Supplementary Fig. S4), had larger starch granules and a tendency toward slightly higher granule numbers (5.0 ± 0.2). The *isa3 lda* double mutant had a stronger starch-excess phenotype (Delatte et al. 2006; Supplementary Fig. S4). Interestingly, its chloroplast sections contained more than triple the number of starch granules compared with the wild type (11.3 ± 4.3). These *isa3 lda* granules ranged widely in size, number, and shape from chloroplast to chloroplast and from cell to cell (see the LM overview in Supplementary Fig. S7).

Transmission electron microscopy (TEM) was used to observe these phenotypes more closely (Fig. 2C). In wild-type chloroplasts, the starch granules appeared in pockets between the thylakoid membranes, with usually one but sometimes more than one granule per pocket. This was also the case in *lda* and *isa3* single mutants. In the *isa3 lda* double mutant, some chloroplasts also had normal-looking starch granules, whereas others had very many small granules per pocket. Occasionally some granules were localized adjacent to the envelope membrane, indicative of ectopic initiation; something not seen in the wild type or the single mutants.

**Figure 3.**
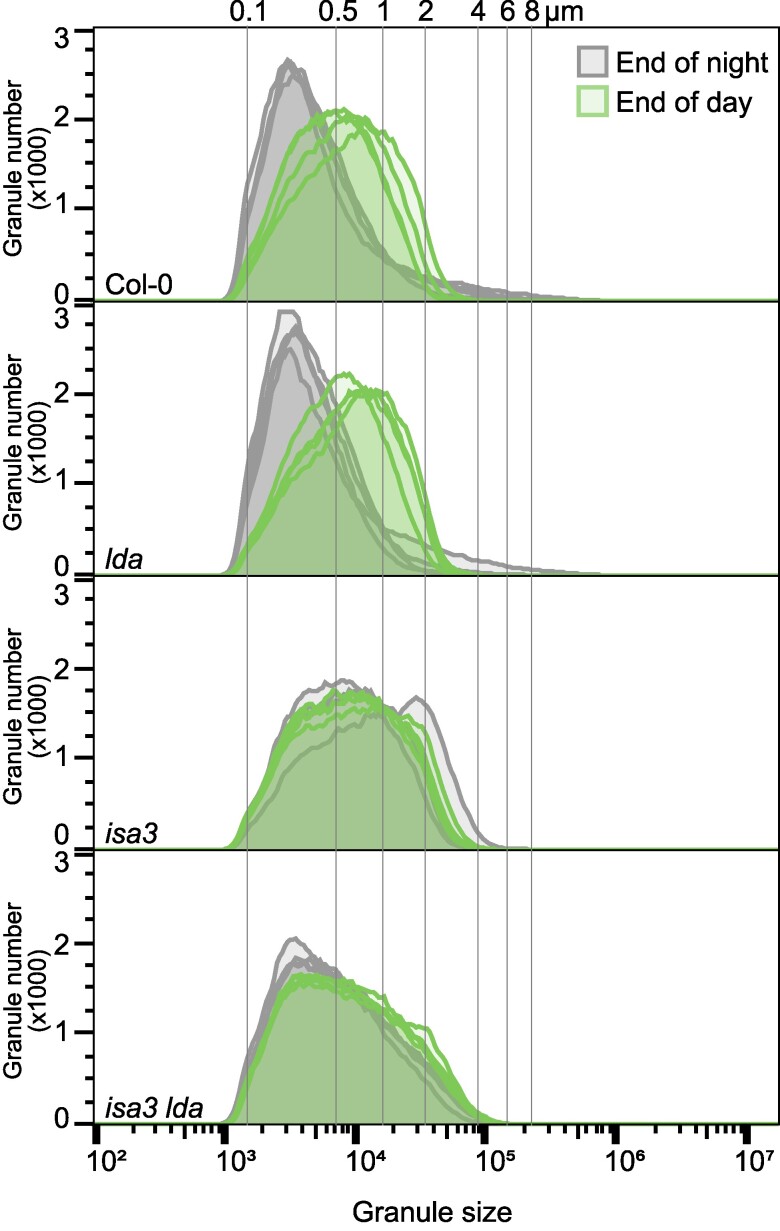
Starch granule size distribution in the absence of ISA3 and LDA. Starch granules were extracted from mature leaves, which were harvested at the indicated times. Size distributions were determined using FC (*n* = 4 biological replicate plants, 100,000 particles measured per replicate).

Analysis of extracted starch granules using flow cytometry (FC) confirmed that both *isa3* and *isa3 lda* exhibited an increase in large granules (2 to 4 *μ*m) compared to the wild type. However, *isa3 lda* also has a high frequency of very small granules (0.1 to 0.5 *μ*m; Fig. 3). Moreover, while wild-type and *lda* starch showed a strong shift toward smaller granules at the end of night, *isa3* and *isa3 lda* starch did not, consistent with their reported deficiency in starch breakdown. Extending this FC analysis to starch extracted from individual leaves of mature rosettes (Supplementary Fig. S8) revealed that older wild-type leaves had larger granules than younger leaves (except for the very oldest, self-shaded leaves). However, the size distribution of the granules in *isa3 lda* remained rather consistent in all leaf samples, being dominated by small granules, even if a slight increase in large granules in the older leaves was also apparent.

The consistent granule size distribution throughout the *isa3 lda* rosette indicates that atypical granule initiation is not a result of starch accumulation. To verify this, we examined the granule number and localization in the youngest leaves with an extended petiole, which have the smallest accumulation of starch (Supplementary Fig. S5). These leaves are the youngest of the young leaf fraction used for starch quantification and MOS analysis (Supplementary Figs. S3 and S4). Even here, *isa3 lda* leaves showed a tendency toward increased granule numbers (4.8 ± 2.6 compared to 3.5 ± 2.0 for the wild type *p*_adj_ = 0.13), with some very small atypical granules apparent (Fig. 2). This suggests that atypical granule initiation begins early in leaf development and that both granule accumulation and the starch-excess phenotype become more pronounced as the leaf ages.

### Increase in granule number is eliminated in the absence of AMY3

To investigate the effect of branched MOS on granule initiation, mutants additionally deficient in AMY3 were used. In the absence of AMY3, soluble branched MOS no longer accumulate, as they are no longer cleaved off from the starch granule surface (Streb et al. 2012; Fig. 1). The activity of AMY3 is increased in *isa3* and *isa3 lda* mutants (Supplementary Fig. S6; Delatte et al. 2006). While *amy3* single mutants did not accumulate excess starch (0.6 ± 0.3 mg/g fresh weight), the absence of AMY3 led to an increase in the starch-excess phenotype in mutants already deficient in starch breakdown (Supplementary Fig. S4). At the end of the night, mature leaves of *isa3*, *isa3 lda*, *amy3 isa3*, and *amy3 isa3 lda* contained increasing amounts of starch (12.9 ± 3.3, 23.2 ± 2.4, 36.9 ± 3.3, and 50.9 ± 6.4 mg/g fresh weight, respectively).

**Figure 4.**
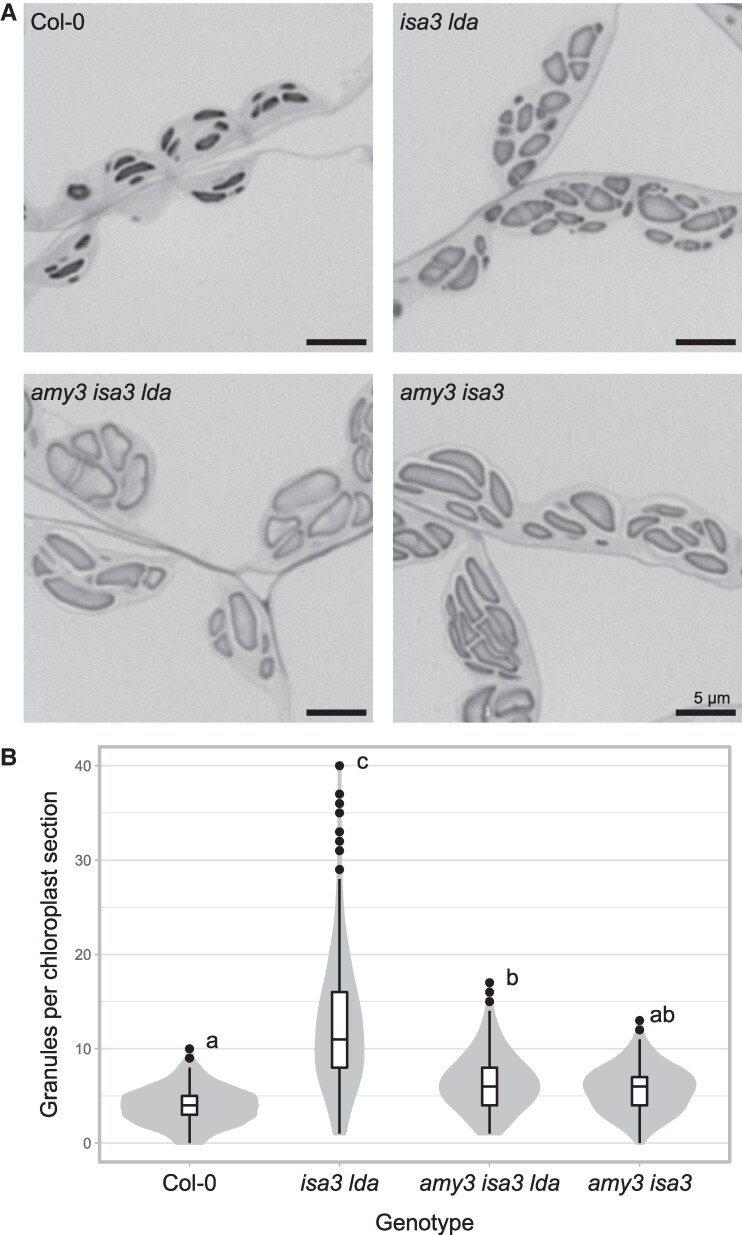
Starch granule number in the absence of AMY3, ISA3, and LDA. **A)** Chloroplasts imaged using LM. Leaf samples were harvested at the end of the light period and stained with Toluidine Blue. Representative images are shown. **B)** The amount of granules visible per chloroplast section in light micrographs was determined for 110 chloroplasts for each of 5 biological replicate plants per genotype. Box plots show median granule number (center line), box limits correspond to the first- and third-quartile. Whiskers extend up to 1.5 times the inter-quartile range from the box limits, points beyond that are plotted individually as outliers. Statistics were done on the biological replicate level. Statistical grouping is based on ANOVA followed by Tukey's Honestly Significant Difference test (*P* < 0.05).

**Figure 5.**
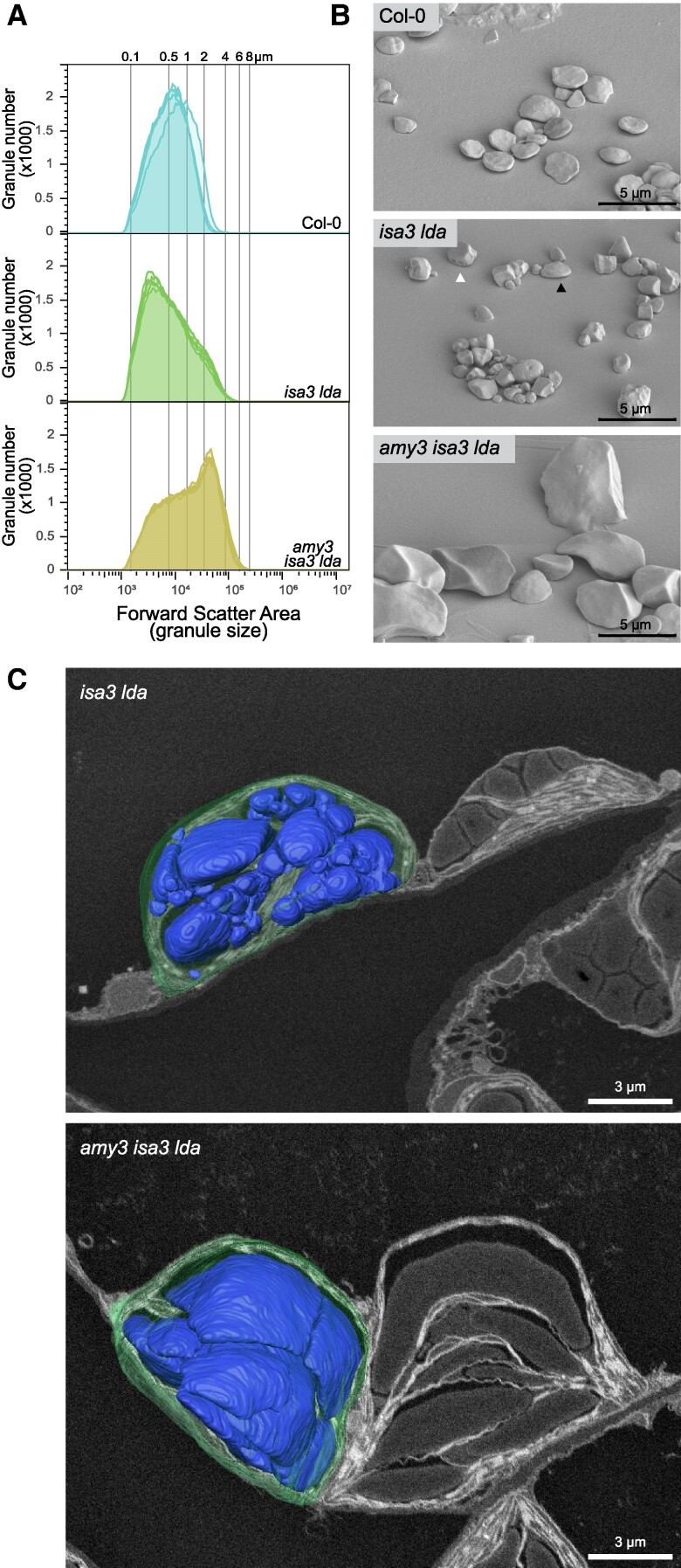
Granule shape and size distribution of *isa3 lda* and *amy3 isa3 lda* at the end of the light period. **A)** Starch granule size distribution of extracted starch as determined by FC (*n* = 5 biological replicate plants, 100,000 particles each). Starch granules were extracted from the mature leaves. **B)** Scanning electron micrographs of extracted starch. For *isa3 lda* the black arrowhead indicates a wild-type-like granule. The white arrowhead indicates a granule with both a polyhedral and an elliptical face. **C)** Serial block-face SEM of entire chloroplasts. Thylakoid membranes (transparent green) and starch granules (blue) were manually annotated and visualized.

Despite its increased starch content, light micrographs revealed significantly fewer granules per chloroplast section in the *amy3 isa3 lda* triple mutant compared to *isa3 lda* (6.41 ± 2.9 vs 12.1 ± 6.2; Fig. 4). The same was true for *amy3 isa3* (5.65 ± 2.4 granules per chloroplast section), which had a starch content more comparable to *isa3 lda* (Supplementary Fig. S4; Streb et al. 2012). FC and scanning electron microscopy (SEM) analysis confirmed this observation: the granule size distribution of *amy3 isa3 lda* was dominated by very large granules, in contrast to the predominance of small granules in *isa3 lda* (Fig. 5). Interestingly, SEM images of *isa3 lda* showed both elliptical wild type-like granules as well as polyhedral granules with sharp faces. The very large granules of *amy3 isa3 lda* appeared to have both convex and concave faces (Fig. 5B). Finally, we used Serial Block-Face scanning electron microscopy (SBF-SEM) to image chloroplasts of both *isa3 lda* and *amy3 isa3 lda* (Supplementary Videos S1 to S3). Image stacks were manually annotated to create a 3D reconstruction of starch granules within a single representative chloroplast, where the high granule number of *isa3 lda* and the high starch content (but lower granule number) of *amy3 isa3 lda* were apparent (Fig. 5C).

Interestingly, our light micrographs suggested that plants accumulating excess starch over their lifetime have larger chloroplasts. This observation was validated using confocal fluorescence microscopy. The chloroplasts of *isa3 lda*, *amy3 isa3*, and *amy3 isa3 lda* showed a significant increase in size compared to either wild type, *isa3* or *amy3* (Supplementary Fig. S9, A and B). The amount of chloroplasts in a fixed volume of palisade cells was approximately halved in these mutants (Supplementary Fig. S9C). Although *isa3* accumulates starch, it did not show a decreased chloroplast number or a significant increase in chloroplast size.

### Absence of canonical granule initiation proteins does not affect over-initiation in *isa3 lda*

**Figure 6.**
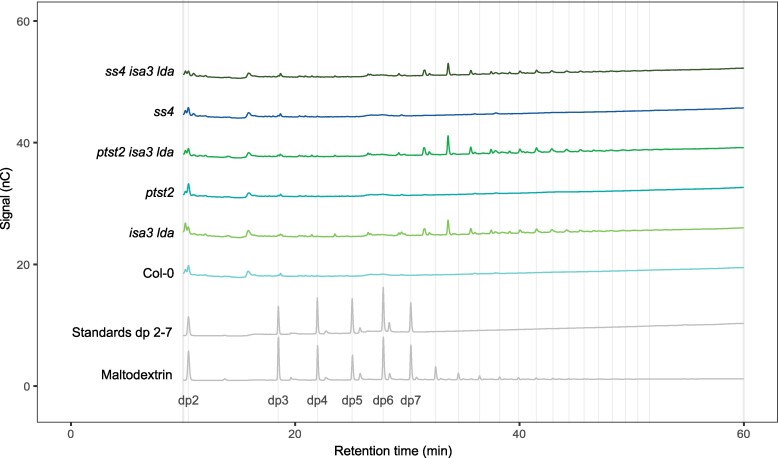
Malto-oligosaccharides of plants deficient in debranching enzymes and initiation proteins. Whole rosettes were harvested at the end of the dark period. Malto-oligosaccharides were extracted and analyzed using HPAEC-PAD. Equal amounts of fresh weight equivalent (2.5 mg) were loaded per sample. Debranched maltodextrin was used to determine the elution times of linear glucans, the first peak shown corresponds to maltose (this chromatogram shown is scaled down to fit the plot). Branched oligosaccharides elute slightly earlier, causing interlinear peaks. Results were consistent across biological replicates (*n* = 4 plants), representative chromatograms are shown (see also Supplementary Fig. S2). The dp 2 to 7 standards have a concentration of 1 *μ*M.

One way for the branched oligosaccharides present in *isa3 lda* to cause over-initiation is their potential use as substrate by the canonical granule initiation system. To test this, we crossed *isa3 lda* plants with plants deficient in SS4 or PTST2, two proteins that are essential for normal granule initiation. HPAEC-PAD analysis confirmed the presence of branched oligosaccharides in extracts of *isa3 lda*, *ptst2 isa3 lda*, and *ss4 isa3 lda* (Fig. 6; Supplementary Fig. S2). As expected, both *ss4* and *ptst2* single mutants did not contain detectable branched oligosaccharides.

**Figure 7.**
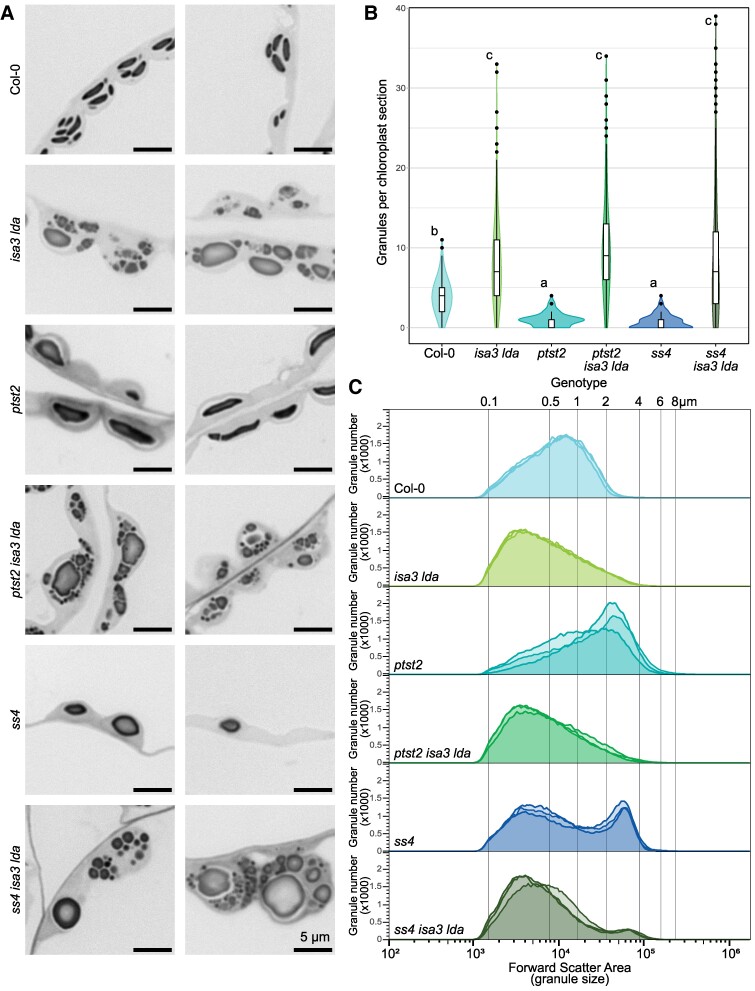
Starch granule number and size distribution at the end of the light period in the absence of debranching enzymes and initiation proteins. **A)** Chloroplasts were imaged using LM. Leaf samples were harvested at the end of the light period and stained with Toluidine Blue. Representative images are shown (see also Supplementary Fig. S7). **B)** Amount of granules visible per chloroplast section in light micrographs. Granule number was determined for 100 chloroplasts for each of 4 biological replicate plants per genotype. Box plots show median granule number (center line), box limits correspond to the first- and third-quartile. Whiskers extend up to 1.5 times the inter-quartile range from the box limits, points beyond that are plotted individually as outliers. Statistics were done on the biological replicate level. Statistical grouping, indicated by letters, is based on ANOVA followed by Tukey's Honestly Significant Difference test (*P* < 0.05). **C)** Starch granule size distribution of extracted starch as determined by FC (*n* = 3 biological replicate plants, 100,000 particles each).

As reported by Seung et al. (2017), the absence of PTST2 causes a strongly reduced granule number compared to wild type (1.0 ± 0.8 respectively 4.0 ± 2.1), as well as substantially larger granules (Fig. 7). However, in the *isa3 lda* mutant background, the absence of PTST2 only causes minor differences; *isa3 lda* and *ptst2 isa3 lda* chloroplasts appeared similar when analyzed by LM (Fig. 7A; additional micrographs in Supplementary Fig. S7). Moreover, *ptst2 isa3 lda* (9.6 ± 5.9) had as many granules per chloroplast section as *isa3 lda* (8.1 ± 5.0; Fig. 7B). FC analysis indicated minimal differences between the size distributions and granule shape of *ptst2 isa3 lda* and *isa3 lda* (Fig. 7C). Furthermore, the distribution of starch between young and mature leaves was similar between *ptst2 isa3 lda* and *isa3 lda* (Supplementary Figs. S4 and S5).

The *ss4* mutant shows a very strong reduction in granule number (0.7 ± 0.8), yet, consistent with our findings with *ptst2*, the high granule number characteristic for *isa3 lda* mutants persists in *ss4 isa3 lda* (8.8 ± 8.2). Interestingly, in contrast to PTST2, the absence of SS4 affects other traits: The triple mutant inherited the round granule shape of *ss4*, displayed a smaller rosette size than either parent, and inherited the pale phenotype of *ss4* (Fig. 7; Supplementary Fig. S3). Furthermore, although the total starch content was similar, the contrast between young and mature leaves was increased (Supplementary Fig. S4). In the *ss4* single mutant, 52% of chloroplast sections observed contained no granules at all. However, in *ss4 isa3 lda*, this number decreased from 52% to 8%. Interestingly, there were large differences in granule size and number between chloroplasts within the same sample (Supplementary Fig. S7). SBF-SEM analysis was employed to investigate whether these variations were due to a sectioning effect. The results revealed that, in contrast to *ss4*, individual chloroplasts within a cell can exhibit a range of granule patterns, including a few large granules, many small granules, or a mixture of both (Supplementary Videos S4 and S5).

## Discussion

### Branched oligosaccharides in *isa3 lda* prime unguided starch granule initiation

The identity and metabolism of oligosaccharide primers is one of the least well-understood aspects of starch granule initiation. Our data suggest that the aberrant branched MOS that accumulate during nighttime starch degradation in *isa3 lda* chloroplasts can serve this primer function, bringing about atypical initiation during daytime photosynthesis and explaining the increased granule numbers seen in this line. We suggest that these branched MOS provide an alternative to the very initial steps in the canonical starch granule initiation process, where long oligosaccharides suitable for the very first branching reactions would be produced by SS4 and its associated proteins.

Several lines of evidence show that the unusually large number of starch granules in *isa3 lda* is caused by unguided initiation events. Firstly, LM analysis of leaf sections of *isa3 lda* reveals an increased granule count per chloroplast specific to this double mutant. In *isa3 lda* two granule populations are apparent—numerous small and relatively few large granules. FC shows that, numerically, the small granule population predominates. We suggest that the large granules are those produced via the canonical starch granule initiation system, while the small granules are atypical, initiated through elaboration of the branched MOS that accumulate specifically in this line. Secondly, this view is supported by the analysis of the *amy3 isa3*, which has a similar starch content as *isa3 lda*, but does not have branched MOS and has no increase in granule number. In this line, fewer MOS will be released into the stroma due to the absence of AMY3, and any that are released can still be catabolized by LDA. Thirdly, the *amy3 isa3 lda* triple mutant, which has the highest starch content of all the lines analyzed, has a far lower granule count than *isa3 lda*. This triple mutant lacks branched oligosaccharides, presumably as none of the remaining starch degradative enzymes are able to release them (Streb et al. 2012). It also lacks the numerous small granules seen in *ias3 lda*, and, although it does have a slightly higher granule count than the wild type, this may be explained by its much higher starch content.

Our comparison of different leaf ages of *isa3 lda* implies that granule over-initiation driven by branched MOS starts early and develops over time. LM and TEM analysis of young leaves already showed a tendency toward an increase in granule number, with the chloroplasts sometimes containing numerous atypical granules. Consistent with this, the FC of extracted starch yielded a similar granule size distribution in all *isa3 lda* leaves, always dominated by small granules (Fig. 2; Supplementary Fig. S8). Unlike starch, branched oligosaccharides do not accumulate with leaf age (Supplementary Figs. S3 and S4). Thus, we propose that granule over-initiation starts in young leaves, and that repeated rounds of atypical initiation during the day combined with impaired starch degradation at night, causes the leaf to accumulate more and more starch granules.

### Over-initiation in *isa3 lda* is not dependent on canonical starch granule initiation machinery

PTST2 and SS4 are both important for the canonical pathway of starch granule initiation proposed for Arabidopsis leaves, and starch granule numbers are sharply reduced in *ptst2* and *ss4* mutants. However, the increased granule number brought about by the loss of ISA3 and LDA is maintained even in the absence of PTST2 or SS4 (Fig. 7). This indicates that the granule over-initiation in *isa3 lda* is independent of these proteins and supports the idea that the branched MOS bypass the canonical initiation pathway and are used as substrates by the other starch biosynthetic enzymes. This is further supported by the aberrant granule localization in *isa3 lda* (Figs. 2 and 5), with some forming in the stroma between the thylakoids and the chloroplast envelope. We have not observed this localization of granules in wild-type plants, where the granules are always observed between the thylakoid membranes. The correct granule localization is controlled by other initiation proteins, particularly the thylakoid localized MFP1, which influences the sub-chloroplastic localization of PTST2 and SS4 proteins (Seung et al. 2018; Sharma et al. 2024).

The *ptst2* and *ss4* mutants both have fewer, larger granules than wild-type plants (Fig. 7). In *ptst2*, the granules are wild-type like in morphology but larger (its starch content is similar to the wild type). Interestingly, there was little difference between the granule size distributions of *ptst2 isa3 lda* and *isa3 lda*. This implies that over-initiation caused by the branched MOS is very influential on the overall final granule distribution, with the large number of atypical small granules overwhelming any difference brought about by PTST2 deficiency on the canonical initiation pathway.

The SS4 protein affects both initiation and the subsequent anisotropic pattern of granule growth (Lu et al. 2018; Bürgy et al. 2021). While most of the starch in the *ss4* mutant is present as a few, large and rounded granules, there is also a population of smaller granules, which can be visualized by SBF-SEM (Bürgy et al. 2021; Supplementary Video S4) or revealed by FC analysis (Fig. 7). The *ss4 isa3 lda* mutant shows an additive phenotype of the *ss4* and *isa3 lda* parental lines; the large round granules typical of *ss4* are still present (Fig. 7), but there are far more small granules, presumably initiated via the branched MOS that accumulate during degradation. Interestingly, these small granules also have the rounded shape characteristic of *ss4* granules, confirming the influence of SS4 on granule growth beyond the initiation stage (Fig. 7; Lu et al. 2018).

Not all *ss4* chloroplasts contain starch, suggesting that SS4-independent initiation events are rare and therefore many chloroplasts may simply fail to initiate any granules (Crumpton-Taylor et al. 2013; Ragel et al. 2013). In the absence of starch, *ss4 isa3 lda* chloroplasts should not generate branched MOS at night and so should not over-initiate granules. Intriguingly, however, the triple mutant had almost no empty chloroplasts (Fig. 7; Supplementary Fig. S7). Additionally, there were distinct granule patterns between chloroplasts within the same *ss4 isa3 lda* cell, with some chloroplasts containing both large and small granules and others containing only small granules (Supplementary Fig. S7 and Video S5). There are several possible explanations for this. For example, upon chloroplast division, daughter chloroplasts could inherit only small granules or branched MOS, but no large granules. Alternatively, MOS could move between chloroplasts through stromules (Hanson and Hines 2018), i.e. from starch containing chloroplasts to starch-free chloroplasts, thereby triggering multiple granule initiations. Such processes could explain both the reduced number of empty chloroplasts, and the distinct granule patterns between chloroplasts in *ss4 isa3 lda*.

### More studies are needed to characterize dynamic MOS metabolism

We still lack important information about the role of MOS in granule initiation, particularly when SS4 is missing. There is evidence that SS3 is primarily responsible for initiation in that case (Szydlowski et al. 2009). However, unlike SS4, there is no evidence that SS3 interacts with the other granule initiation proteins, i.e. PTST2 and MFP1, that are proposed to collectively bind suitable MOS with high affinity at predetermined sites of initiation (Seung et al. 2017, 2018; Sharma et al. 2024). The simplest explanation could be that SS3 and BEs act on soluble MOS in the stroma, and some of these products undergo transition to an insoluble phase (Liu et al. 2023). If so, any factors that influence the concentration and/or nature of the MOS pool could potentially influence SS4-independent initiation. For example, we previously proposed that AMY3 interferes with such initiation events, potentially by endo-amylolytic cleavage of longer MOS chains. The evidence for this was indirect, based on the observation that removal of AMY3 activity in *ss4* mutants led to an increase in granule numbers (Seung et al. 2016).

It is important to consider that MOS, as well as being intermediates of starch degradation, may also be produced in other ways. MOS may be synthesized de novo, and a pathway where maltose is produced via a cytosolic heteroglycan intermediate has been suggested (Merida and Fettke 2021). MOS are also by-products of starch synthesis within the plastid, liberated as amylopectin is trimmed by ISA1/ISA2 DBE into an optimal structure for crystallization (Ball et al. 1996). Indeed, a recent study on the role of the phosphorylase PHS1 in starch granule initiation in the wheat endosperm suggested that it may elongate MOS trimmed from existing granules, thereby producing primers to support new granule initiation events (Kamble et al. 2023). Other studies of phosphorylase function in rice (*Oryza sativa*) endosperm also suggest a role in production of primers for starch synthesis (Satoh et al. 2008; Dong et al. 2023).

MOS metabolism in the plastid stroma requires further careful study. The MOS pool is likely to be highly dynamic, with continuous elongation and degradation by different enzymes, even if the levels and the absolute fluxes are relatively low. An understanding of these dynamics will be needed to establish the relationship between MOS and starch granule initiation, and it will be crucial to determine exactly which MOS species are utilized as primers and what happens at the point when their elaboration successfully seeds a new granule.

Here, we used *isa3 lda* double mutant's deficiency in starch breakdown as a tool to investigate the role of MOS in granule initiation. However, it is important to note that MOS over-accumulation can also have consequences for the chloroplast. For instance, swollen or ruptured chloroplasts were reported in plants such as *mex1* mutants (deficient in the chloroplast envelope maltose transporter, MALTOSE-EXCESS 1), where maltose produced during starch breakdown accumulates in the stroma, leading to an osmotic imbalance with the cytosol (Stettler et al. 2009; Cho et al. 2011; Malinova et al. 2014). We did not observe any evidence for a loss of chloroplast integrity in *isa3 lda*, and the amounts of glucan accumulating as branched MOS were much less than accumulates as maltose in *mex1* (Delatte et al. 2006; Stettler et al. 2009). However, we did observe a reduced number of chloroplasts in *isa3 lda* (Supplementary Fig. S9). This appears to be associated with starch accumulation rather than MOS, since a similar reduction was observed in *amy3 isa3* and *amy3 isa3 lda*. Furthermore, the accumulation of starch and reduction of chloroplast number correlated with an increase in chloroplast size, either due to the volume of starch itself or because its constant presence impedes chloroplast division.

### Over-initiation in *isa3 lda* leads to compound starch granules

Recently it was shown that, during de novo granule initiation in destarched wild-type chloroplasts, multiple initials are generated within the same stromal pocket during the first minutes of the day (Bürgy et al. 2021). ^13^CO_2_-labelling and NanoSIMS analyses revealed that many of these initials then coalesce to become one starch granule within the first hour of synthesis, after which the granules grow anisotropically. It is unclear what controls coalescence, but it was suggested to occur during the very first stages of granule initiation, possibly in a stromal micro-environment defined by the initiation proteins. Growing granules that subsequently abut continue to grow on adjacent surfaces, apparently without fusing, possibly because once granules get beyond a certain size, fusion does not occur (Bürgy et al. 2021).

A notable feature of the *isa3 lda* chloroplasts is that they contain many small abutting starch granules that presumably grow without fusing (Figs. 2 and 5). The resulting granules are reminiscent of polyhedral-shaped granules from rice seed endosperm starch, where the granules also have flat abutting faces, and rounded external surfaces (Jane et al. 1994). It is presently unclear if, or to what extent, the *isa3 lda* granules that are initiated from the branched MOS in the stroma coalesce like those generated by the granule initiation apparatus. If they do coalesce, then the extent of over-initiation in *isa3 lda* would be even higher than reported here. Alternatively, granules primed by branched MOS might not coalesce, perhaps because components of the initiation apparatus are needed for coalescence or because they are initiated relatively far from each other in the stroma and come into contact after exceeding the suggested size threshold for fusion. It is interesting, however, that in the *ss4 isa3 lda* triple mutant, all granules lost their polyhedral-like morphology and appear more spherical, despite being formed together in the same stromal pockets (Supplementary Video S5). This re-emphasizes that granule growth, whether between the thylakoid membranes or next to an adjacent granule is a directed process for which SS4, and presumably other protein factors are required.

Further work will be needed to address some of the important outstanding questions surrounding starch granule initiation and growth, both in Arabidopsis, where electron tomography coupled with ^13^CO_2_-labelling and NanoSIMS studies have proven to be useful tools (Bürgy et al. 2021), but also in storage tissues of crop plants, where starch granule number, shape and size influence functional properties of starch (Li et al. 2023).

## Materials and methods

### Plant materials and growth conditions

All *Arabidopsis* (*A. thaliana*) mutants were in the Columbia-0 (Col-0) background. The single mutant *amy3-2* (SAIL_613D12) was described by Yu et al. (2005). The mutants *isa3-2* (GABI_280G10), *lda-2* (SALK_060765), and the double mutant *isa3-2 lda-2* were described in Delatte et al. (2006). The double and triple mutants *amy3-2 isa3-2* and *amy3-2 isa3-2 lda-2* were generated and described by Streb et al. (2012). Finally, *ss4-1* (GABI_290D11) was described by Roldán et al. (2007), and *ptst2-7* (SALK_73591) by Seung et al. (2018). Genotypes of all lines were confirmed using PCR. The triple mutants *ss4 isa3 lda* and *ptst2 isa3 lda* were obtained by crossing and selecting homozygous triple mutant plants from segregating F2 populations. Selection was done by PCR-based genotyping using primers described in Supplementary Table S1.

Seeds were stratified on soil in darkness at 4 °C for 3 d. Plants were grown on soil in individual pots in growth cabinets at 60% relative humidity, temperature of 20 °C and 150 *μ*mol m^−2^ s^−1^ of light with a 12-h light/12-h dark diel cycle. Plants were harvested after 35 d, immediately before the start of the dark or light period. The first leaf with a fully extended petiole and the largest leaf were designated as respectively young and mature.

### Enzyme activities

Activities of BEs and SSs were determined as described in Pfister et al. (2016). The same protocol was used for DPE2 and phosphorylase activities; samples were loaded on a 7.5% acrylamide gel containing 0.2% oyster glycogen (Merck). After electrophoresis, gels were washed and incubated in buffer containing 100 mm citrate-NaOH (pH 6.5) for 4 h at 37 °C in presence of 20 mm maltose (For DPE2) or 20 mm glucose-1-phosphate (for phosphorylase). For DPE1 activities, the protein extracts were incubated for 20 min at 37 °C in buffer containing 50 mm NaAcetate and 7 mm maltotriose. Control samples were boiled before incubation. Glucose production was determined using the enzyme-linked assay described in Hostettler et al. (2011).

### FC analysis of starch granules

Starch granules were extracted and analyzed according to Thieme et al. (2023). Briefly, measurements were performed on a CytoFLEX 6 flow cytometer calibrated using sizing beads with a similar optical density to starch. The lower limit of detection was 0.1 μm. For FC analysis of starch from individual rosette leaves, 2 plants with the same size and leaf number were used. From one, starch was extracted from each leaf separately and analyzed FC. The other was used to create the reference photograph.

### Light and electron microscopy

Samples from the leaf lamina (2 × 2 mm) were cut from both young and mature leaves of 35-d-old rosettes, fixed in 2.5% (v/v) glutaraldehyde; 2% (w/v) formaldehyde in 0.1 m sodium cacodylate (pH 7.4), stained with 2% (w/v) osmium tetroxide and embedded in Spurr resin (Polysciences) using a Pelco BioWave Pro+ tissue processing microwave (Ted Pella; Abt et al. 2020). For LM, semi-thin (0.5 μm) sections of the embedded leaf were stained with Toluidine Blue O and imaged using an AxioImager Z2 microscope (Zeiss). Images were taken with a 40× or a 100× oil-immersion lens. Visible starch granule sections were counted for 100 chloroplast sections per biological replicate, exclusively from palisade cells. Cells adjacent to vasculature were excluded. For TEM, thin (70 nm) sections of the embedded leaf were stained with 2% (w/v) uranyl acetate and Reynold's lead citrate and imaged at 2,500× magnification using a Morgagni 268 (Thermo Fischer Scientific). For chloroplast volume and number analysis, chlorophyll fluorescence of fresh leaf material was imaged using a Zeiss LSM 780 AxioObserver confocal microscope. The excitation wavelength used was 633 nm at 5% intensity, and emission was detected at 647 to 721 nm with a detector gain of 750. Chloroplasts were counted in *Z*-stacks of 106 × 106 × 14.7 µm. Chloroplast diameters were measured in FIJI at the *z*-level where the chloroplasts were the largest. Embedding and staining for SBF-SEM was performed as described by Bürgy et al. (2021). After curing at 60 °C, samples were trimmed and glued on pins. SBF-SEM was performed using a FEI Apreo VolumeScope (Thermo Fisher Scientific) operated at 1.18 kV. Image stacks were annotated and analyzed using AMIRA software (Thermo Fisher Scientific). For SEM, extracted starch granules were adhered on poly-L-lysin precoated silicon wafer chips for 15 min. After 3 dips in distilled water, the chips were allowed to air dry. The dried chips were mounted on SEM stubs with silver paint and finally sputter coated with 4 nm Pt/Pd in a Safematic CCU-010 metal sputter coater. Images were taken in Magellan 400 FEI SEM at 2 kV by secondary electron detection.

### Starch staining with I_2_/KI solution

Whole rosettes were decolorized in 80% (v/v) ethanol, rinsed with water, and stained with Lugol solution consisting of 10% (w/v) potassium iodide and 5% (w/v) iodine (Sigma-Aldrich). Excess stain solution was washed away with water and the rosettes photographed.

### Extraction and analysis of soluble malto-oligosaccharides and starch

Unless otherwise specified, entire rosettes were weighed and snap-frozen in liquid N_2_. Alternatively, rosettes were split into young and mature leaves, excluding the very youngest leaves (those without extended petioles) and the cotyledons. In this case, each biological replicate consisted of either young or mature leaves pooled from 4 plants. Samples were homogenized and extracted in cold 0.7 m HClO_4_ using an all-glass homogenizer. The homogenized material was separated by centrifugation (10 min, 3,000 × *g*, 4 °C) into soluble and insoluble fractions. The insoluble fraction, containing starch, was washed once with water, 5 times with 70% (v/v) ethanol and resuspended in 1 ml water. Starch quantification was performed as described by Hostettler et al. (2011). The soluble fraction, containing soluble MOS, was neutralized to pH 6 by adding 2 m KOH, 0.4 m KCl, and 0.4 m MES. Precipitated potassium perchlorate was removed by centrifugation (10 min, 3,000 × *g*, 4 °C) and the resultant extracts were stored at −20 °C until use.

For the analysis of MOS in the soluble fraction, samples with and without enzymatic debranching treatments were compared. Where specified, 3 volumes of methanol were first added and samples incubated at −20 °C overnight as a precaution to precipitate any starch solubilized during tissue homogenization. The soluble fraction was collected and methanol was removed by centrifugal evaporation. An aliquot of the sample was digested using 0.08 U/ml *Pseudomonas* isoamylase (Megazyme) and 2 U/ml *Klebsiella planticola* pullulanase (Megazyme) in 10 mm sodium acetate buffer, pH 4.8 for 4 h at 37 °C. Enzymes were deactivated by boiling for 10 min and removed by centrifugation (10 min, 4,000 × *g*, 20 °C). Nondigested samples were treated the same, but without enzyme addition. Maltodextrin produced from corn starch (Sigma-Aldrich) was debranched as above and used as control to determine the elution times of linear glucans.

Prior to HPAEC-PAD analysis, ions in the samples were exchanged using sequential Dowex 50 and Dowex 1 columns (Sigma-Aldrich) and concentrated by freeze-drying, as described by Hostettler et al. (2011). HPAEC-PAD was performed using a Dionex ICS-5000 (ThermoFisher) equipped with a 50 mm guard column and a 250 mm analytical column (PA200 IC; ThermoFisher) with a flow rate of 0.4 ml min^−1^ and the following program: 0–7 min, eluent A (100 mm NaOH); 7 to 55 min, linear gradient to 45% A, 55% B (500 mm NaAcetate, 150 mm NaOH); 55 to 65 min, linear gradient to 15% A, 85% B; 65 to 65.5 min, linear gradient to 100% A; 65.5 to 80 min, 100% A.

### Research data

Research data presented in this work are available in Supplementary Data Set 1.

### Accession numbers

Relevant sequence information related to this article can be found under the AGI codes mentioned in Supplementary Table S1.

## Supplementary Material

kiaf002_Supplementary_Data

## Data Availability

The data underlying this article are available in the article and in its online supplementary material.

## Supplementary data

The following materials are available in the online version of this article.

**Supplementary Figure S1.** Debranched malto-oligosaccharides extracted from *isa3 lda* plants

**Supplementary Figure S2.** Malto-oligosaccharides in plants deficient in starch breakdown enzymes and initiation proteins.

**Supplementary Figure S3.** Malto-oligosaccharides in the young and mature leaves of plants deficient in starch breakdown.

**Supplementary Figure S4.** Starch content in the young and mature leaves of plants deficient in starch breakdown and/or initiation at the end of the dark period.

**Supplementary Figure S5.** Starch accumulation at the end of the light or dark period in plants deficient in starch breakdown and/or initiation.

**Supplementary Figure S6.** Enzyme activities related to starch and oligosaccharide biosynthesis in plants deficient in starch breakdown.

**Supplementary Figure S7.** Light micrographs of plants deficient in starch breakdown enzymes and initiation proteins.

**Supplementary Figure S8.** FC of starch isolated from individual rosette leaves of Col-0 and *isa3 lda* plants.

**Supplementary Figure S9.** Chloroplast volume and number in mutants deficient in starch breakdown enzymes.

**Supplementary Video S1.** Starch granules in the chloroplasts of Col-0 imaged using SBF-SEM.

**Supplementary Video S2.** Starch granules in the chloroplasts of *isa3 lda* plants imaged using SBF-SEM.

**Supplementary Video S3.** Starch granules in the chloroplasts of *amy3 isa3 lda* plants imaged using SBF-SEM.

**Supplementary Video S4.** Starch granules in the chloroplasts of *ss4* plants imaged using SBF-SEM.

**Supplementary Video S5.** Starch granules in the chloroplasts of *ss4 isa3 lda* plants imaged using SBF-SEM.

Supplementary video legends brief description and relevant details of **Supplementary Videos S1 to S5.**

**Supplementary Table S1.** Oligonucleotide primers used to genotype T-DNA lines.

**Supplementary Data Set 1.** Raw data acquired in this study.

## Dive Curated Terms

The following phenotypic, genotypic, and functional terms are of significance to the work described in this paper:

ISA3 Gramene: AT4G09020
ISA3 Araport: AT4G09020

DPE2 Gramene: AT2G40840
DPE2 Araport: AT2G40840

PHS1 Gramene: At3g29320
PHS1 Araport: At3g29320

SEX4 Gramene: AT3G52180
SEX4 Araport: AT3G52180

ISA1 Gramene: AT2G39930
ISA1 Araport: AT2G39930

MOS CHEBI: CHEBI:17593

starch CHEBI: CHEBI:28017

AMY3 Gramene: AT1G69830
AMY3 Araport: AT1G69830

SS4 Gramene: AT4G18240
SS4 Araport: AT4G18240

MFP1 Gramene: AT3G16000
MFP1 Araport: AT3G16000

PTST2 Gramene: AT1G27070
PTST2 Araport: AT1G27070

LDA Gramene: AT5G04360
LDA Araport: AT5G04360
