# Supplementary material for: Branched oligosaccharides cause atypical starch granule initiation in Arabidopsis chloroplasts
Source: Plant Physiol. 2025 Jan 9;197(2):kiaf002. doi: 10.1093/plphys/kiaf002 (PMC11809589; doi:10.1093/plphys/kiaf002)
Supplement: kiaf002_Supplementary_Data [file kiaf002_supplementary_data.zip › PP2024RA00998R1_Supplementary_Video_Legends.docx]

**Supplementary Video S1.** Starch granules in the chloroplasts of the Col-0 imaged using SBF-SEM. The video shows chloroplasts of wild-type Arabidopsis at the end of the light period. The field of view is approximately 26 x 26 μm. Note that these chloroplasts show a similar number of starch granules, which are separated by thylakoid membranes.

**Supplementary Video S2.** Starch granules in the chloroplasts of *isa3 lda* plants imaged using SBF-SEM. The video shows chloroplasts of Arabidopsis deficient in both ISA3 and LDA at the end of the light period. The field of view is approximately 26 x 26 μm. Note the large number of granules, which are often present within the same stromal space.

**Supplementary Video S3.** Starch granules in the chloroplasts of *amy3 isa3 lda* plants imaged using SBF-SEM. The video shows chloroplasts of Arabidopsis deficient in AMY3, ISA3 and LDA at the end of the light period. The field of view is approximately 26 x 26 μm. Note that these chloroplasts have a much smaller increase in granule number compared to *isa3 lda*, despite having more starch, and that most stromal spaces have only one granule.

**Supplementary Video S4.** Starch granules in the chloroplasts of *ss4* plants imaged using SBF-SEM. The video shows chloroplasts of Arabidopsis deficient in SS4 at the end of the light period. The field of view is approximately 26 x 26 μm. Note that most chloroplasts have one or several large round starch granules, as well as a number of small granules, whereas some have none at all.

**Supplementary Video S5.** Starch granules in the chloroplasts of *ss4 isa3 lda* plants imaged using SBF-SEM. The video shows chloroplasts of Arabidopsis deficient in SS4, ISA3 and LDA at the end of the light period. The field of view is approximately 26 x 26 μm. Note the large differences in granule phenotype between chloroplasts. Some have a number of very large round granules separated by thylakoid membranes, whereas others have high numbers of very small granules within the same stromal space.
