# Supplementary material for: Branched oligosaccharides cause atypical starch granule initiation in Arabidopsis chloroplasts
Source: Plant Physiol. 2025 Jan 9;197(2):kiaf002. doi: 10.1093/plphys/kiaf002 (PMC11809589; doi:10.1093/plphys/kiaf002)
Supplement: kiaf002_Supplementary_Data [file kiaf002_supplementary_data.zip › Supplementary materials.pdf]

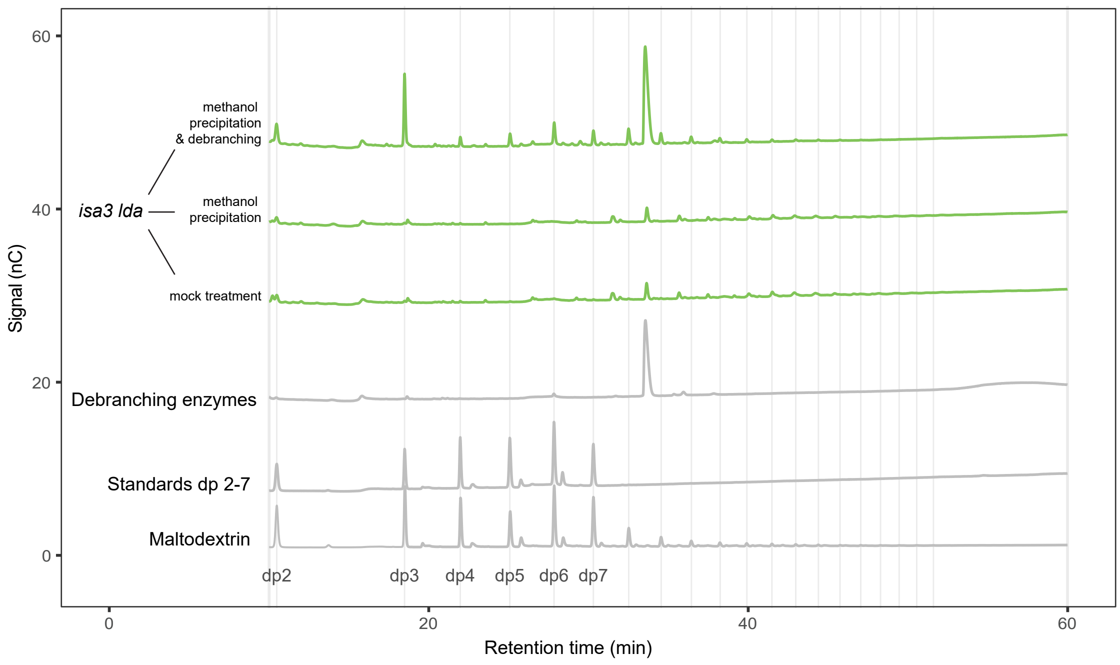

Supplementary Figure S1. Malto-oligosaccharides of plants deficient in ISA3 and LDA. Whole rosettes were harvested at the end of the dark period. Malto-oligosaccharides were extracted and analyzed using HPAEC-PAD. Equal amounts of fresh weight equivalent (2.5 mg) were loaded per sample. Samples were subjected to methanol precipitation in order to remove any solubilized amylopectin and subsequently debranched using isoamylase and pullulanase. Debranched maltodextrin was used to determine elution times of linear glucans, the first peak shown corresponds to maltose (this chromatogram shown is scaled down to fit the plot). Branched oligosaccharides elute slightly earlier, causing interlinear peaks. The degree of polymerization (dp) 2-7 standards have a concentration of 1 µM.

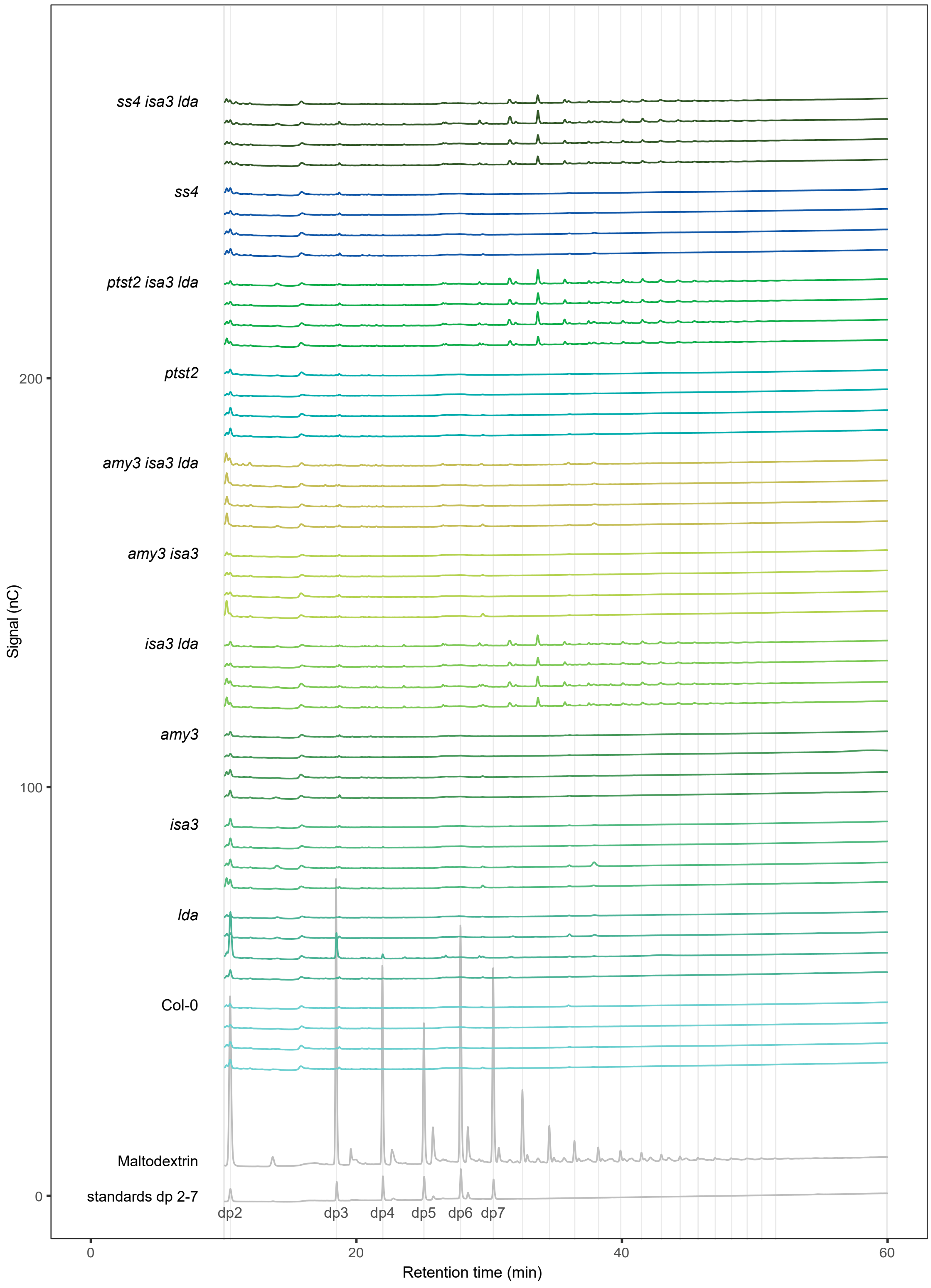

Supplementary Figure S2. Malto-oligosaccharides of plants deficient in starch breakdown enzymes and initiation proteins. Whole rosettes were harvested at the end of the dark period. Malto-oligosaccharides were extracted and analyzed using HPAEC-PAD. Equal amounts of fresh weight equivalent (2.5 mg) were loaded per sample. Debranched maltodextrin was used to determine elution times of linear glucans, the first peak shown corresponds to maltose. Branched oligosaccharides elute slightly earlier, causing interlinear peaks. Results were consistent across replicates (n=4 plants). The degree of polymerization (dp) 2-7 standards have a concentration of 1 µM.

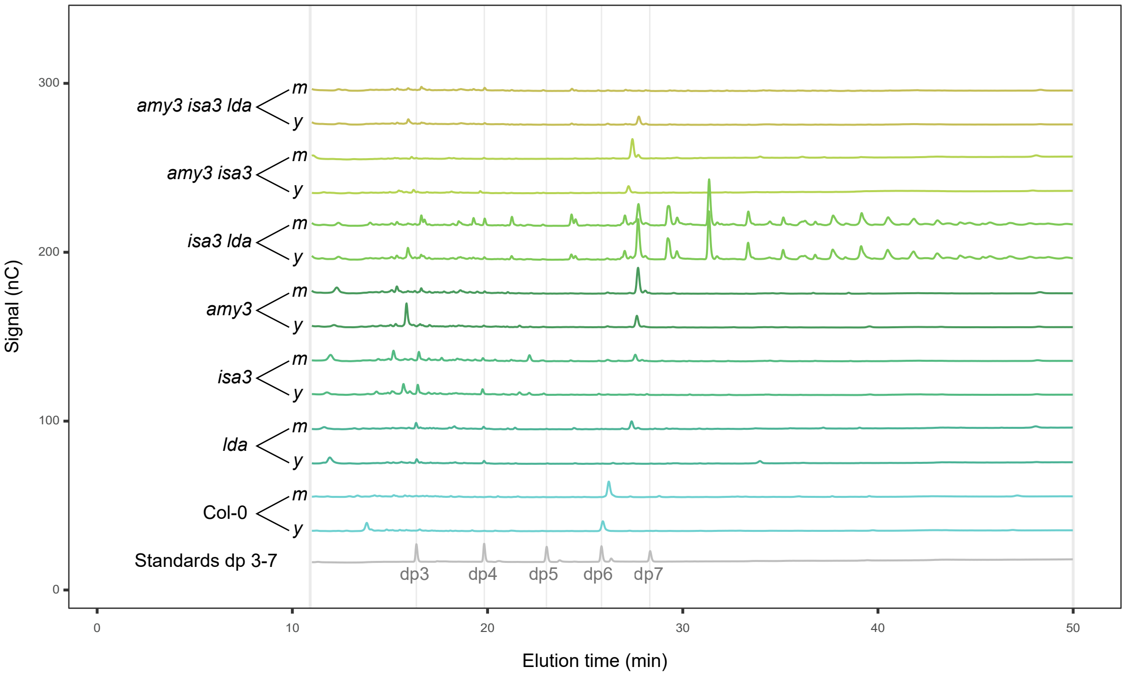

Supplementary Figure S3. Malto-oligosaccharides in the young and mature leaves of plants deficient in starch breakdown. Soluble sugars extracted from young (y) and mature (m) leaves were harvested at the end of the dark period and analyzed using HPAEC-PAD. Equal amounts of fresh weight (12.5 mg) were loaded per sample. Linear glucan standards with degree of polymerization (dp) 3-7 (1μM) were used to determine the retention times of linear glucans. Results were consistent across biological replicates (n=4 plants), representative chromatograms are shown.

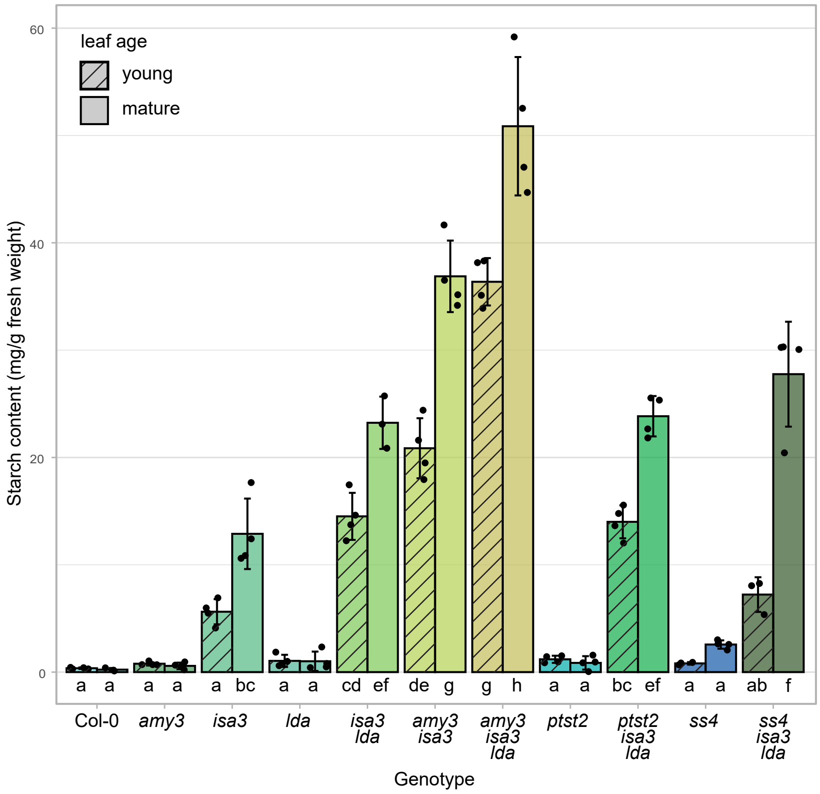

Supplementary Figure S4. Starch content in the young and mature leaves at the end of the dark period. Young and mature leaves were harvested separately. Starch was extracted and quantified using an enzyme-linked assay after complete hydrolysis to glucose. Error bars represent standard deviation (n=4 biological replicate plants). Statistical grouping shown on the horizontal axis is based on ANOVA followed by Tukey's HSD test (p<0.05).

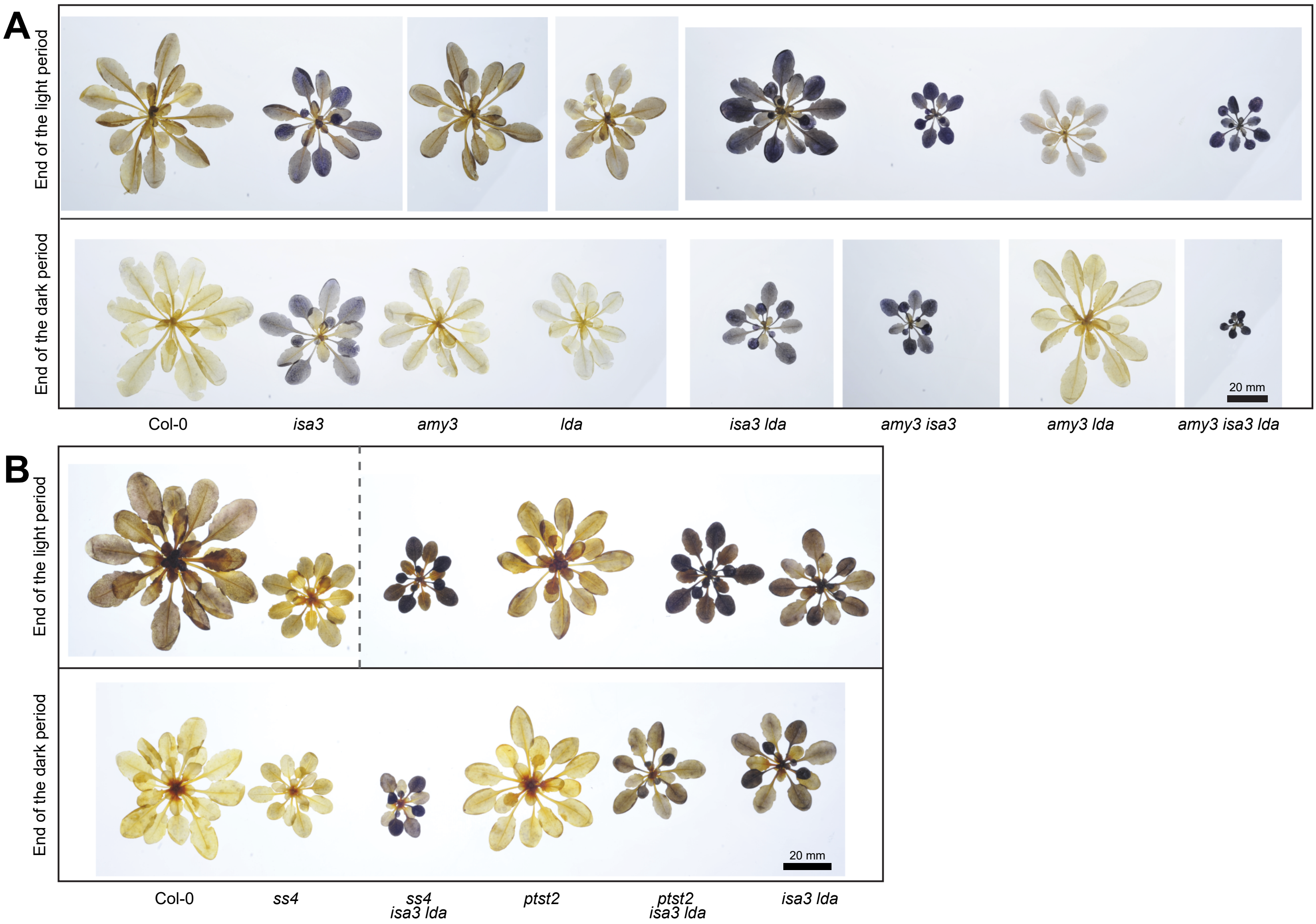

Supplementary Figure S5. Starch accumulation at the end of the light or dark period. Rosettes were cleared in 80% ethanol and stained using Lugol's solution. (A) Starch accumulation and distribution in the absence of starch breakdown enzymes. Photographs were taken at the same time, the same magnification and under the same light conditions. (B) Starch accumulation and distribution in the absence of debranching enzymes and initiation proteins. Photographs were taken at the same time, the same magnification and under the same light conditions. The dashed line indicates the border between two photographs.

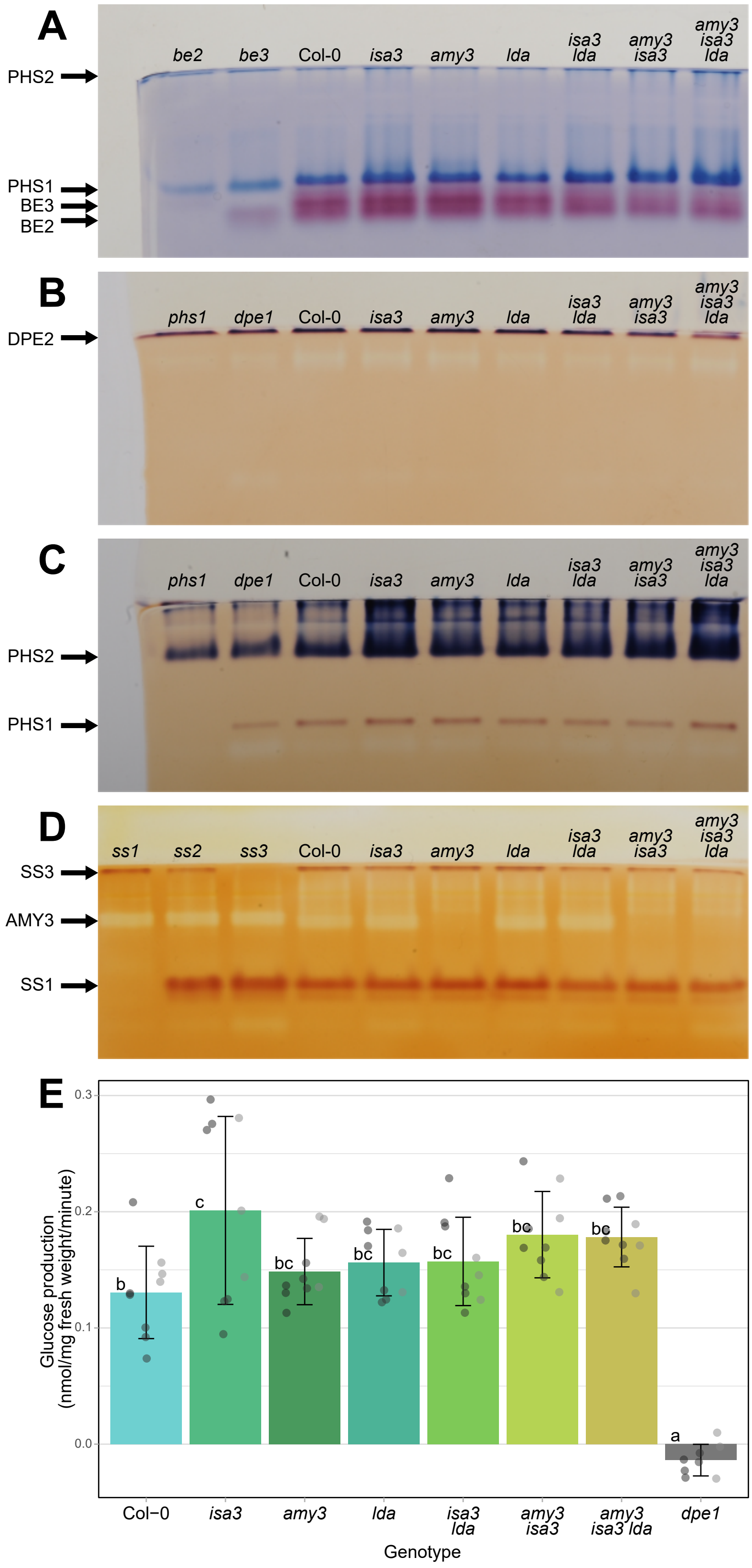

Supplementary Figure S6. Enzyme activities related to starch and oligosaccharide biosynthesis in plants deficient in starch breakdown. 15 microgram of protein was loaded per well. (A) Native gel showing activities of BRANCHING ENZYME 2 and 3, and PHOSPHORYLASE 1 and 2. Representative gel is shown (n=5 biological replicates). (B) Native gel showing activity of DISPROPORTIONATING ENZYME 2 (DPE2). Representative gel is shown (n=3 biological replicates). (C) Native gel showing activities of PHOSPHORYLASE 1 and 2. Representative gel is shown (n=3 biological replicates)). (D) Native gel showing activities of STARCH SYNTHASE 1 and 3, as well as α-AMYLASE 3. Representative gel is shown (n=5 biological replicates). (E) Activity of DISPROPORTIONATING ENZYME 1 (DPE1) determined by incubation with maltotriose, followed by the quantification of produced glucose. Technical replicates are grouped (n=3 biological replicates). Significance groups are based on ANOVA followed by Tukey's HSD test ($p<0.05$). Error bars indicate standard deviation.

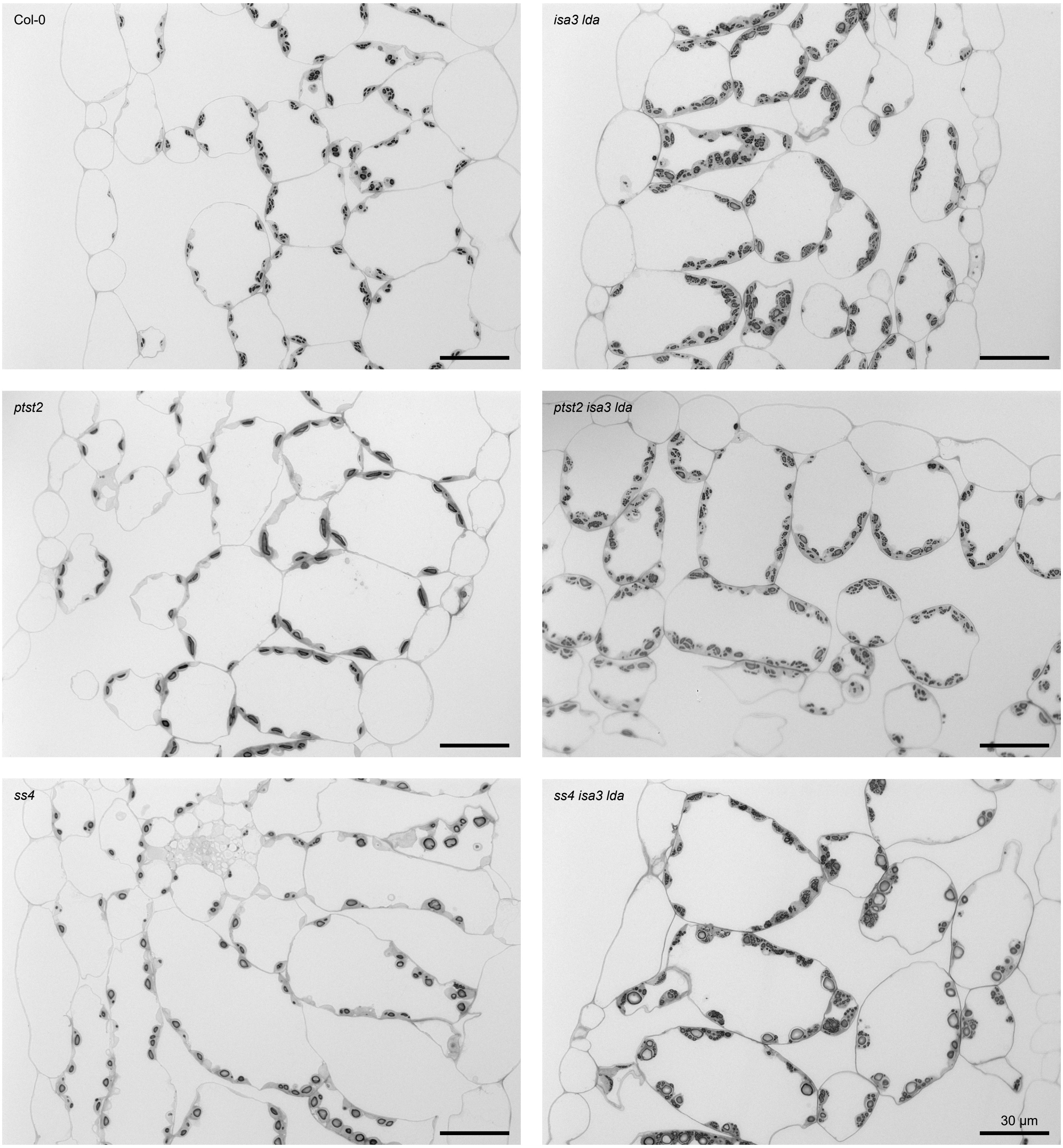

Supplementary Figure S7. Light micrographs of plants deficient in starch breakdown enzymes and initiation proteins. Mature leaf samples were harvested at the end of the light period and stained with toluidine blue. Representative images are shown (n=4 biological replicates).

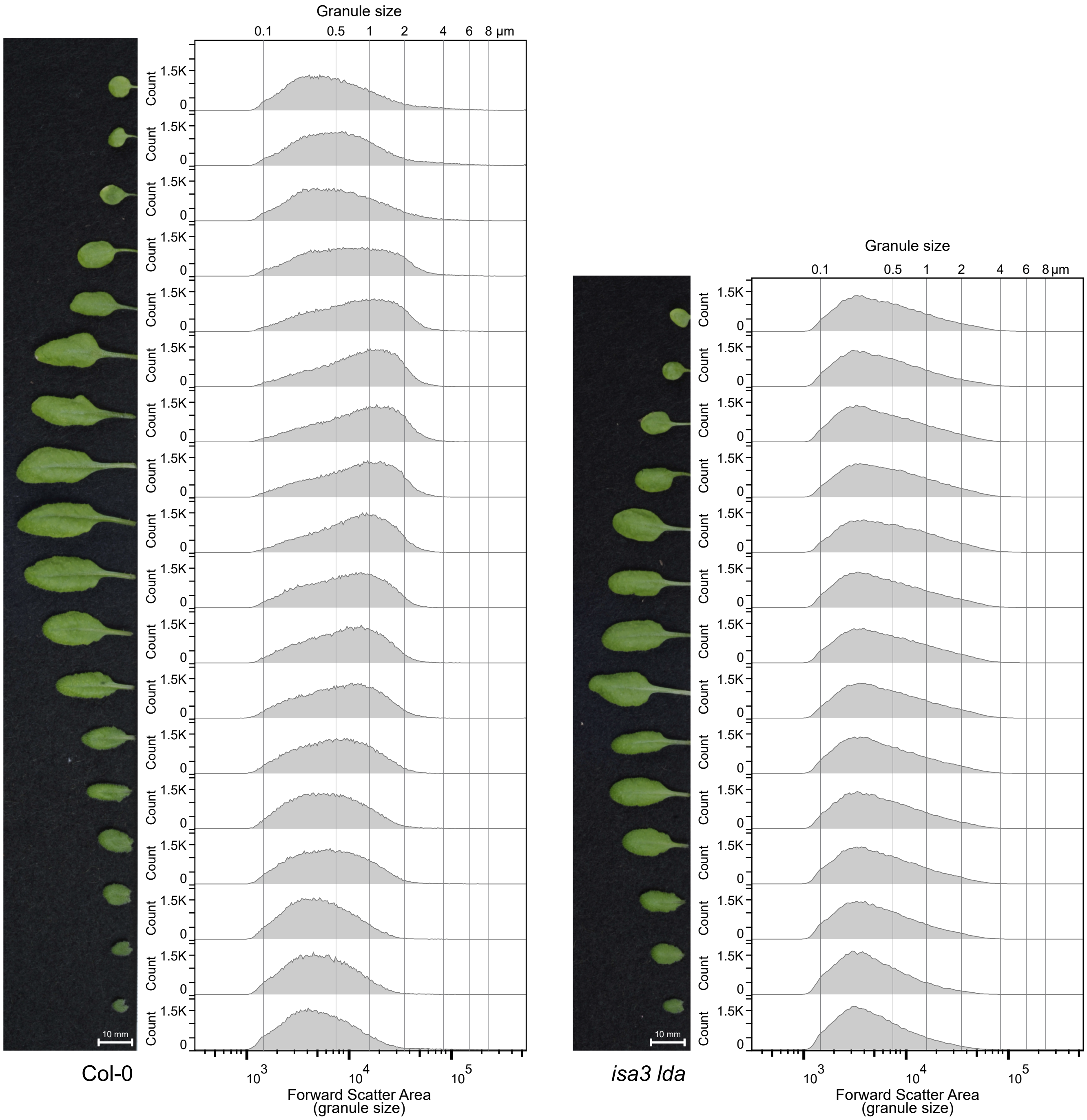

Supplementary Figure S8. Rosette flow cytometry of Col-0 and *isa3 lda* plants. Starch was extracted from each individual leaf of a rosette and the granule size distribution of 100,000 granules each was determined using flow cytometry. Representative leaves are shown to the left of the histograms. Results were consistent among 3 experimental replicates, of which one is shown.

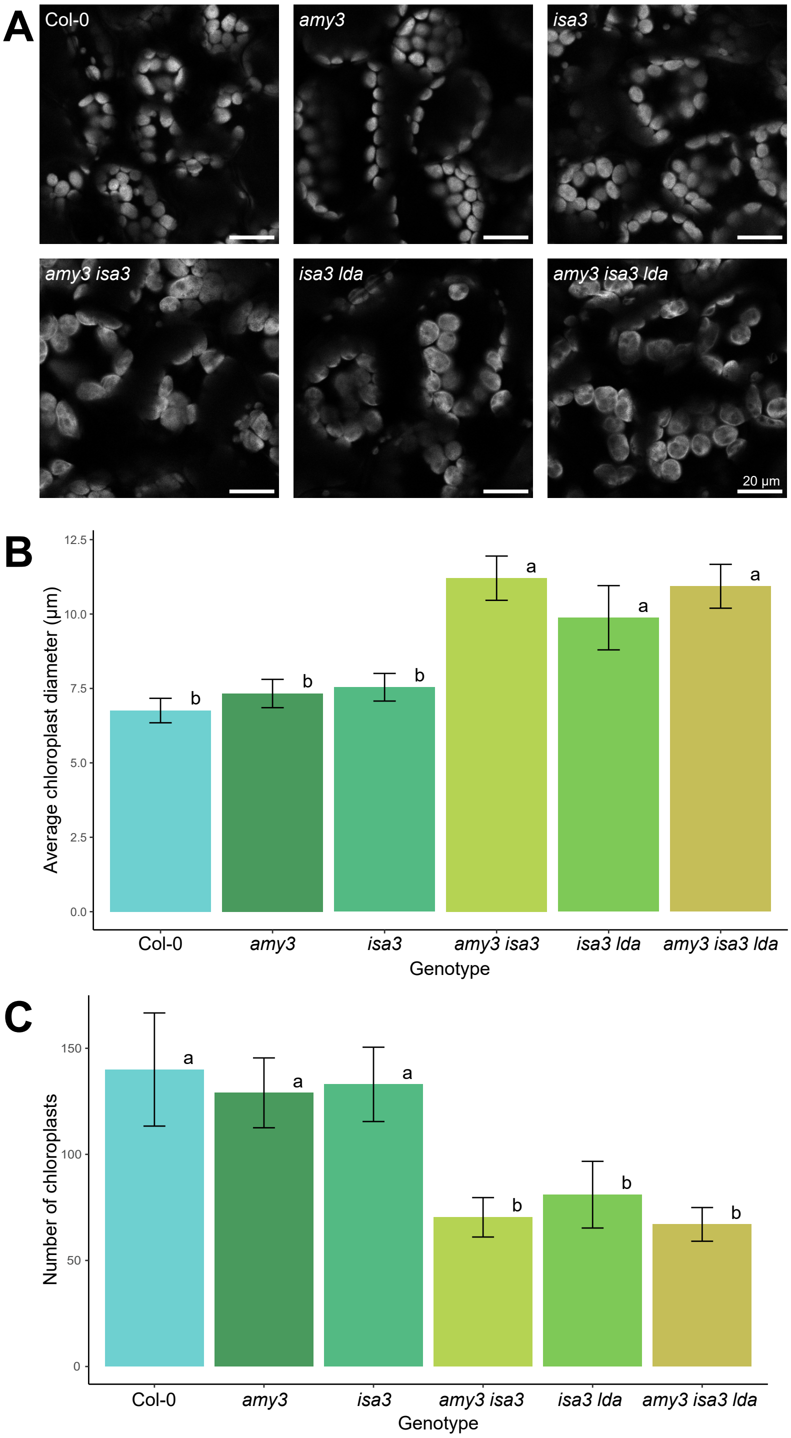

Supplementary Figure S9. Chloroplast volume and number in mutants deficient in starch breakdown enzymes. (A) Chlorophyll autofluorescence detected using confocal microscopy of palisade cells. (B) Average length of the largest diameter of chloroplasts. Statistics were done on the biological replicate level, values represent mean± sd (n=3 biological replicate plants, at least 45 chloroplasts were measured per plant). Significance groups are based on ANOVA followed by Tukey's HSD test (p<0.05). (C) Number of chloroplasts present in a fixed imaged volume (106*106*14.7 µm). Statistics were done on the biological replicate level, Values represent mean ± sd (n=3 biological replicate plants). Significance groups are based on ANOVA followed by Tukey's HSD test (p<0.05).

Mutant	Line	Gene	Locus	Fw primer	Rv primer	T-DNA primer
ss4-1	GABI_290D11	ss4	AT4G18240.1	TGTTCATCTCTCTTTCTCCT	CAATACCTTCAAATTCCCTC	ATATTGACCATCATACTCATTGC
ptst2-7	SALK_73591	ptst2	AT1G27070	GCCTTTTCAGAAATGGATAAATAGCCTTGCTTCC	GAGACAACTGCTGGGAACTTG	TGGTTCACGTAGTGGGCCATCG
amy3-2	SAIL_613D12	amy3	AT1G69830	CCGACCTTGTGAAATTTCTTCACTG	GGTTCCTCTTGTAGACGATGTTCC	GCCTTTTCAGAAATGGATAAATAGCCTTGCTTCC
isa3-2	GABI_280G10	isa3	AT4G09020	GGAGAAGGAGGAAATGATG	GGATTGAAGAGAATGGGG	CCCATTTGGACGTGAATGTAGACA
lda-2	SALK_060765	lda	AT5G04360.1	TTGTAGTTGGGGAGGATG	GGAAGAGAGGAAGATAATTGG	TGGTTCACGTAGTGGGCCATCG

Supplementary Table S1. Primers used in genotyping T-DNA insertion mutants. The intact genes were amplified using the Fw and Rv primer. T-DNA inserts were amplified with the T-DNA and Rv primer
