# Supplementary material for: Branched oligosaccharides cause atypical starch granule initiation in Arabidopsis chloroplasts
Source: Plant Physiol. 2025 Jan 9;197(2):kiaf002. doi: 10.1093/plphys/kiaf002 (PMC11809589; doi:10.1093/plphys/kiaf002)
Supplement: kiaf002_Supplementary_Data [file pp.00998.2024-s12.pdf › pp.00998.2024-s12.pdf]

Plant Physiology®

Branched oligosaccharides cause atypical starch granule initiation in Arabidopsis chloroplasts

Arvid Heutinck, Selina Camenisch, Michaela Fischer-Stettler, Mayank Sharma, Barbara Pfister, Simona Eicke, Chun Liu, and Samuel Zeeman

Review Timeline:

Submission Date:	24-Jun-2024
Editorial Decision:	24-Jul-2024
Revision Received:	25-Oct-2024
Accepted:	25-Nov-2024

24-Jul-2024

Prof. Dr. Samuel C Zeeman
Eidgenossische Technische Hochschule Zurich
Zurich CH-8092
Switzerland

RE: Branched oligosaccharides cause spontaneous starch granule initiation in Arabidopsis chloroplasts

Dear Dr. Zeeman:

We have received reviews of your manuscript, which you may view at the end of this letter. On the basis of the advice received and my own evaluation, I believe that, pending revision, this article may ultimately be acceptable for publication in Plant Physiology.

Both reviewers requested editing of the manuscript. I am unsure this needs additional experiments, but I encourage the authors to do their best to revise their manuscript according to all the comments raised by the reviewers, including performing new experiments when needed. In particular:
1) L118: Please clarify whether the soluble sugar fraction contains phytoglycogen or not. You may already have the data?
2) L121, Figures 1, 6, and Figure S1: Please include at least three biological replicates to show variability between the samples in the supplementary figures.
3) Please compare the chain length distribution pattern of starch fractions and also analyze the size of amylopectin molecule.

When you are ready to submit your revision, click the below link and find the red arrow under your Author Tasks heading for this revision submission.

If you want to visit this version of the manuscript, you may click the link below and then click on the folder that reads "Post Decision Manuscripts".

Link Not Available

When uploading the revised version of this article, please be sure to include as complete an answer as possible to the referees' criticisms during the first round of reviews in the "response to reviewers" section.

Plant Physiology considers a revision for up to 60 days after a manuscript has been provisionally accepted; limited extension of this timeframe is at the discretion of the Monitoring Editor. A reminder notice will be sent to you 2 weeks before the deadline, at which time you may choose to contact the editorial office to request an extension; if so, please provide a justification for your request. After the expiration of this deadline, the article would need to be submitted as a resubmission and would be handled as such.

Thank you very much for giving us an opportunity to review this work. I look forward to receiving the next version.

Sincerely,

Editor, Plant Physiology

Want to add this revision deadline to your calendar? Click below!

--- Reviewer comments:
Reviewer #1 (Comments for the Author):

This manuscript describes the possibility that branched MOS (maltooligosaccharides) produced during starch degradation could serve as primers for the initiation of starch granules formation in Arabidopsis leaves. To this end, specific mutant lines affected for starch degradation were used: the single isa3 mutant defective for the main starch debranching enzyme (an isoamylase) involved in starch degradation and the double mutant isa3 lda, lda being the pullulanase-type debranching enzyme. Then,

mutation for either SS4 or PTST2, two proteins of the starch pathways demonstrated to be involved in the priming of starch synthesis in Arabidopsis leaves, were combined to isa3 lda and the impact on starch content, granule's structure and number was assessed. While granules number per chloroplast was strongly reduced in the ptst2 and ss4 mutants, it was not the case when these mutations were combined with isa3 lda. Granules number was strongly increased in isa3 lda compared to wild type and remained as high when ptst2 or ss4 mutation was added.
This is an interesting piece of work and results that brings additional information about the process of starch initiation that is still not fully understood.

Comments:

1) It is not clear to me what is presented in Figs 1, 6 and S1. Are those chromatograms of fully debranched MOS? Were linear and branched MOS separated before debranching and application to HPAEC-PAD?
2) I would appreciate having data about the amount of MOS (branched or not) accumulating in the different lines used in this work. Also, since it is stated that branched MOS seem only involved in the initiation of starch granules, I find it useful to give data about the relative amount of linear and branched MOS in the different lines.
3) Line 408 p. 12: I do imagine that "insoluble malto-oligosaccharides" refers to starch. Also "soluble malto-oligosaccharides" refers to linear or branched MOS. Is that correct? Please clarify.
4) What is the source of maltodextrins such as used in Fig. 1? Same for "glucans dp 1-7" in Fig. S1.
5) I don't understand exactly why the amount of starch granules is only slightly higher in young leaves of the isa3 lda double mutant when it seems (to be confirmed as requested in point 2 above) that the amount of branched MOS is equivalent to that reported in mature leaves. If these branched MOS could interfere with the 'canonical' priming process, we would expect there to be many small starch granules, which is not the case (Fig. 2B). Starch overaccumulation is already very high in young isa3 lda leaves (Fig. S2), so the impact should already be substantial. I am not entirely convinced by the explanation given in the discussion.
6) I suggest that the activity of other starch-metabolizing enzymes should be checked in the different lines studied. As suggested in the discussion, MOS (branched or linear) could appear in different ways. I suggest checking the activity of starch phosphorylases and glucanotransferases (DPE1/DPE2).

Reviewer #2 (Comments for the Author):

This manuscript revealed absence of both isoamylase (ISA) and pullulanase (LDA) increases number of starch granules and contents of starch and MOS in Arabidopsis leaf. Additional loss of amylase (AMY3) mitigates the phenotype observed by the double mutant thus claiming MOS is key substance for initiating starch granules.

The reviewer has several comments which would likely to be addressed.
Title: This manuscript claims that branched MOS cause "spontaneous" starch granule initiation. However, the reviewer does not see the results associated with "spontaneous" granule initiation.
The author should also consider that presence of branched MOS at the end of the dark period and increased number of starch granules can be separate cause. For examples, when debranching enzymes are absent, it is likely excess branches are generated by branching enzymes. Thus, amylopectin molecule cannot become any larger due to steric hinderance. Analysis of triple mutant, isa lda be2, should be performed to clarify whether balance between branching and debranching influence the granule initiation and generation of branched MOS. The reason why isa lda amy3 triple mutant showed a similar phenotype to the WT can be that loss of debranching enzymes, amy3 did not degrade starch, thus amylopectin branch could be longer and was not affected by the osmotic pressure.

L118: Please clarify whether the soluble sugar fraction contains phytoglycogen or not.
L121, Figures 1, 6, and Figure S1: The elution patterns of debranched MOS extracted from isa3 lda are different between Fig1 and FigS1, particularly the peak between DP 6 and 7. Please include at least three biological replicates to show variability between the samples in the supplementary figures.
Please compare the chain length distribution pattern of starch fractions and also analyze the size of amylopectin molecule.
Also please explain the reason why the peaks are not aligned to the maltodextrin standard. If the elution pattern of debranched and non-debranched samples are compared, then it will be clear 1) which peak is from liner MOS, 2) which contains short branch residue, 3) which peak has modification (such as phosphate etc).

L155: Please consider the possibility that the increase in starch granule number associated with the growth stage may suggest involvement of other starch biosynthetic enzymes, which has different spatio-temporal expression patterns.

L177: Please analyze the chain length distribution pattern of amylopectin and amylose content of the small and large starch granules fractionated by flow cytometry to show the differences in the composition of starch granules.

We very much appreciate the interest and the constructive comments/suggestions of both reviewers. Below is a point by point response.

Reviewer 1:

COMMENT 1: It is not clear to me what is presented in Figs 1, 6 and S1. Are those chromatograms of fully debranched MOS? Were linear and branched MOS separated before debranching and application to HPAEC-PAD?

RESPONSE: Fig. 1, Fig. 6 and Fig. S1 (now Fig. S3) show chromatograms of undebranched MOS. Separation into linear and branched MOS before HPAEC-PAD analysis is not feasible. We identify peaks as branched oligosaccharides based their elution slightly earlier than linear oligosaccharides of the same size, and their disappearance upon enzymatic debranching *in vitro* (see Fig. S1 and Streb et al. 2012). The wild-type and mutant plants studied here do not accumulate measurable amounts of linear oligosaccharides in the size range that we measured (dp3 until ~25), presumably as they are metabolized by β-amylases and other enzymes. We have edited the manuscript to state this in the results.
We repeated the experiments presented in Fig. 1 and Fig. 6 (see also the point 5 by Reviewer 2) and included methanol precipitation and debranching to show conclusively that the peaks represent branched oligosaccharides (Fig. S1). We found that methanol precipitation was necessary when debranching samples since extraction with the glass homogenizers can solubilize small amounts of starch, from which linear chain fragments are released by debranching (now stated in the Materials and Methods).
In *isa3 lda* samples, most branched oligosaccharide peaks disappear upon debranching, although some smaller peaks are resistant (Fig. S1), likely because they contain short glucosyl stubs that are poor substrates for the commercial debranching enzymes used. These peaks disappear after amyloglucosidase treatment, confirming them to be MOS (not shown).

COMMENT 2: I would appreciate having data about the amount of MOS (branched or not) accumulating in the different lines used in this work. Also, since it is stated that branched MOS seem only involved in the initiation of starch granules, I find it useful to give data about the relative amount of linear and branched MOS in the different lines.

RESPONSE: It is possible to quantify the peak areas that are present in these chromatograms. However, translating these peak areas to molar values requires several assumptions. Branched oligosaccharides standards are not available. Furthermore, they can have different configurations, especially as the dp increases. We can calibrate the PAD detector for known concentrations of linear dp1-7 glucan standards. As shown in Fig. S3, the detector is more sensitive towards smaller glucans. Calibration standards with a higher dp tend to be impure. Regardless, a crude quantitative analysis of the branched glucan peaks of *isa3 lda* shows a content of between 0.25 and 0.41 mg/g FW, which we believe to be a slight underestimation. Indeed, Delatte et al (2006), determined the amount of soluble glucans in *isa3 lda* by using the enzyme-linked assay used for determining starch content, finding a content of ~1 mg/g FW soluble MOS (now stated in the Introduction). These could not be precipitated using 75% (v/v)

methanol, 1% (w/v) KCl, indicating low molecular weight MOS. As mentioned above, the plants studied here do not accumulate linear oligosaccharides.

COMMENT 3: Line 408 p. 12: I do imagine that "insoluble malto-oligosaccharides" refers to starch. Also "soluble malto-oligosaccharides" refers to linear or branched MOS. Is that correct? Please clarify.

RESPONSE: This was indeed unclear and we corrected the wording in the manuscript to starch. Soluble malto-oligosaccharides refers to both linear and branched MOS.

COMMENT 4: What is the source of maltodextrins such as used in Fig. 1? Same for "glucans dp 1-7" in Fig. S1.

RESPONSE: Maltodextrin derived from corn starch was purchased from Sigma-Aldrich and debranched using *Pseudomonas* isoamylase and *Klebsiella planticola* pullulanase. Glucans dp1-7 contains equimolar glucose, maltose, maltotriose, maltotetraose, maltopentaose, maltohexaose, and maltopentaose. We have renamed this to reflect the glucan standards that are visible in the chromatogram. We have included this information in the Materials and Methods section.

COMMENT 5: I don't understand exactly why the amount of starch granules is only slightly higher in young leaves of the isa3 lda double mutant when it seems (to be confirmed as requested in point 2 above) that the amount of branched MOS is equivalent to that reported in mature leaves. If these branched MOS could interfere with the 'canonical' priming process, we would expect there to be many small starch granules, which is not the case (Fig. 2B). Starch overaccumulation is already very high in young isa3 lda leaves (Fig. S2), so the impact should already be substantial. I am not entirely convinced by the explanation given in the discussion.

RESPONSE: Upon reflection, it was not clear enough in the manuscript that 'young leaves' are defined differently in both experiments. When determining starch and branched MOS accumulation, rosettes were split into younger leaves (excluding the very smallest leaves without extended petioles) and more mature leaves (excluding the cotyledons). Thus, this 'young' sample consists of the younger half of all leaves. In the light and electron microscopy analysis, it is the youngest leaf with an extended petiole, i.e. the youngest of the young leaves that was analyzed. This is clearer in the revised manuscript, when we compare the results of these experiments. Additionally, we put more emphasis on Fig. S8 (Rosette FCM), which supports the idea that over-initiation starts in the young leaves, and granules accumulate throughout the leaf's lifetime.

COMMENT 6: I suggest that the activity of other starch-metabolizing enzymes should be checked in the different lines studied. As suggested in the discussion, MOS (branched or linear) could appear in different ways. I suggest checking the activity of starch phosphorylases and glucanotransferases (DPE1/DPE2).

RESPONSE: We agree with this suggestion and have determined the activity of a number of enzymes and included this in the manuscript. Some lines exhibit small increases in Phosphorylase 2 and Disproportionating Enzyme 1 (DPE1) activity, and/or small decreases in Starch Synthase 1 and 3, as well

as Branching Enzymes 2 and 3 (Now stated in the Results and shown in Fig. S6). We recognize that these changes will likely affect maltooligosaccharide metabolism. However, as the changes in enzyme activities of *isa3 lda* and *amy3 isa3 lda* were similar, we feel it is unlikely that they explain the presence of branched MOS specifically in *isa3 lda*. Thus, we favor the explanation that the branched MOS found in *isa3 lda* are likely to be produced through starch breakdown rather than the action of starch phosphorylases and glucanotransferases. The phosphorylases and glucanotransferases may well play a role in the extension of these branched MOS during the establishment of the atypical granules.

Reviewer 2:

COMMENT 1: Title: This manuscript claims that branched MOS cause "spontaneous" starch granule initiation. However, the reviewer does not see the results associated with "spontaneous" granule initiation.

RESPONSE: We agree that the word “spontaneous” is not the most accurate description of what is happening and think “atypical” is more accurate. The title has been changed.

COMMENT 2: The author should also consider that (the) presence of branched MOS at the end of the dark period and increased number of starch granules can be separate cause. For examples, when debranching enzymes are absent, it is likely excess branches are generated by branching enzymes. Thus, amylopectin molecule cannot become any larger due to steric hindrance.

RESPONSE: In the mutants studied here, the ISA1/2 isoamylase involved in amylopectin maturation is still present. In *Arabidopsis*, this is sufficient to trim excess branches and prevent steric hinderances that could result in the formation of phytoglycogen. The difference in function between ISA1/2, ISA3 and LDA is related to their substrate preferences. ISA1/2 acts on longer chains, which are present during starch synthesis, whereas ISA3 and LDA prefer shorter chains (e.g. maltosyl and maltotriosyl stubs produced by β-amylase during starch breakdown; Streb et al., 2012). Unlike *isa1 isa2* mutants, the *isa3 lda* double mutant does not accumulate phytoglycogen during the day. Rather, the amount of soluble glucans in *isa3 lda* decreases during the light and increases during the dark period, consistent with them being products of starch breakdown (Delatte et al., 2006).

COMMENT 3: Analysis of triple mutant, isa lda be2, should be performed to clarify whether balance between branching and debranching influence the granule initiation and generation of branched MOS. The reason why isa lda amy3 triple mutant showed a similar phenotype to the WT can be that loss of debranching enzymes, amy3 did not degrade starch, thus amylopectin branch could be longer and was not affected by the osmotic pressure.

RESPONSE: Arabidopsis has two active branching enzymes: BE2 and BE3 (both Class-2 branching enzymes), which are functionally redundant. There is no Class-1 branching enzyme. In absence of both BE2 and BE3, no starch is produced. Therefore, we don’t expect the suggested triple mutant (or rather the quadruple mutant deficient in both of the Class-2 branching enzymes) to yield informative results (the BE mutations will be epistatic). Previously, the amylopectin chain length distributions of *isa3*, *isa3*

lda and *amy3 isa3 lda* were analysed. All were very similar with a small increase in very short chains and a slight decrease in medium length chains (Streb et al., 2012) due to the loss of ISA3. Loss of AMY3 did not alter this pattern. This makes it unlikely for the differences in branch length to affect starch granule initiation. We are not sure we fully understand the reviewer's reference to an osmotic effect.

COMMENT 4: L118: Please clarify whether the soluble sugar fraction contains phytoglycogen or not.

RESPONSE: Please see response to point 2, above.

COMMENT 5: L121, Figures 1, 6, and Figure S1: The elution patterns of debranched MOS extracted from isa3 lda are different between Fig1 and FigS1, particularly the peak between DP 6 and 7. Please include at least three biological replicates to show variability between the samples in the supplementary figures.

RESPONSE: The reviewer is right that there was variation between datasets and appreciate this critical comment. We believe this was caused by technical rather than biological variation. The data in Fig. 1 and Fig. 6 in the last version of this paper was collected by using 12.5 mg fresh weight equivalent per run. Although this increases the size of the branched oligosaccharide peaks, it also increases the matrix effects on elution times due to salts introduces during sample preparation steps. We have redone these chromatography experiments with a newly extracted set of samples using 2.5 mg fresh weight equivalent per run, resulting in strongly increased reproducibility between replicates (see new Fig. S2). We updated Fig. 1 and Fig. 6 with the new data.

COMMENT 6: Please compare the chain length distribution pattern of starch fractions and also analyze the size of amylopectin molecule.

RESPONSE: Please see response to point 3, above. We did not analyze the amylopectin size, although it could be combined with analyzing the properties of the small and large granule populations in a follow-on study (see below in the response to Point 9).

COMMENT 7: Also please explain the reason why the peaks are not aligned to the maltodextrin standard. If the elution pattern of debranched and non-debranched samples are compared, then it will be clear 1) which peak is from linear MOS, 2) which contains short branch residue, 3) which peak has modification (such as phosphate etc).

RESPONSE: Please see response to Reviewer 1, point 1.

Other modifications could shift the retention time of glucans. We don't think that there are phosphate groups on the glucans (the only *in-vivo* modification to our knowledge) as these greatly increase the retention time. Additionally, phosphoglucans would be removed during our sample preparation steps.

COMMENT 8) L155: Please consider the possibility that the increase in starch granule number associated with the growth stage may suggest involvement of other starch biosynthetic enzymes, which has different spatio-temporal expression patterns.

RESPONSE: Please see response to reviewer 1-6.

COMMENT 9) L177: Please analyze the chain length distribution pattern of amylopectin and amylose content of the small and large starch granules fractionated by flow cytometry to show the differences in the composition of starch granules.

RESPONSE: This is an interesting idea. Biochemical insight on the difference between large and small granules would help in answering questions related to starch initiation and establishment. In our case, there is a good chance that there is not so much difference between the chain length distribution of the two samples based on previous data (considering the small difference between *isa3 lda* and *amy3 isa3 lda*). However, this type of experiment would be very suitable for follow-up work, along with the suggestion to look at the amylopectin molecular size.

25-Nov-2024
Prof. Dr. Samuel C Zeeman
Eidgenossische Technische Hochschule Zurich
Zurich CH-8092
Switzerland

MSID: PP2024-RA-00998R1
MS TITLE: Branched oligosaccharides cause atypical starch granule initiation in Arabidopsis chloroplasts

Dear Sam:

I am pleased to inform you that your revised manuscript "Branched oligosaccharides cause atypical starch granule initiation in Arabidopsis chloroplasts" has been accepted for publication in Plant Physiology. Your manuscript will be checked for consistency with journal formatting and image standards by one of our Science Editors, who will contact you shortly. They may have a number of queries and suggestions for you. Please keep in mind that they work to increase the online visibility of your article and its attraction for readers from the widest of backgrounds. You will have the opportunity to review any revisions, and I hope you will work constructively with them to get the most out of your research. You will also have the opportunity to review all changes when you receive the author proofs for your article.

Once validated for publication, your article will be sent to a Science Editor for a final review of your manuscript and source files. After your final files are uploaded, your article will appear in the next available issue and the online Preview version of your article will be posted after your signed Copyright and Checklist forms and final source files are received.

Please be sure to include the following in your manuscript file:
Harvard style citations
List of all author contributions and funding information at the beginning of manuscript after the titles and author list.
List all supplemental materials associated with the article, with titles, at the end of the ms, before the references and acknowledgments section.
Complete Figure Legends for all figures appearing in the article listed before the Literature Cited.

LICENSE INFORMATION Shortly after your paper is sent to production, you will receive an email from our publisher's author support team, **SciPris**, with information on article processing charges, as well as the types of Open Access license you would be able to purchase. The accepted version of your manuscript will not appear on our Advance Articles page until the license is selected and signed, so please look out for an email from SciPris. For more information see the Article Publication Fees section of our author guidelines: https://academic.oup.com/plcell/pages/General-Instructions

Finally, we encourage your submission of artwork for the journal cover. If there is an image or illustration related to your paper that you would like to have considered, please email it in .pdf or .tif format to clowe@aspb.org. An image title, brief, 2-3 sentence description of the image, and the name of the person credited for the image are also encouraged. If your image is selected to appear on the online cover of the journal, you will be contacted with the scheduled issue date and further information.

Thank you and congratulations on your Plant Physiology paper!

Sincerely,
Pierdomenico

Editor, Plant Physiology

=====
FIRST AUTHOR PROFILES
=====

We recognize the hard work that goes into being First Author of a Plant Physiology paper by publishing a "First Author Feature" for each First Author alongside your paper.

If the first author(s) would like to submit information for a feature on Facebook and Twitter, fill out the form by clicking on the link

below:

Plant Physiology First Author Feature Form: https://forms.office.com/r/ZBCGZfhm8F
If you cannot access the form through the link above, please contact Rachel at rbelsky@aspb.org.

To upload an image of yourself to accompany your profile, please put your photo in the correct journal folder at the link below. You must label your image: Author First Name Last Name Article Title.

First Author Profile Image Dropbox: https://www.dropbox.com/scl/fo/xz029p7qjr2jml2rp2kmq/h?dl=0&rlkey=6fkxcaqmxx35ap152b2osy1fz

=====
IMPORTANT REMINDER: PEER REVIEW REPORTS
=====

If you opted to publish a peer review report along with your article during the original submission process, it will be prepared by the editorial staff and publicly posted with your manuscript, inside the zip file that contains any other supplemental material. As a reminder, the peer review report is a public record of all comments from editors and reviewers, as well as your prior responses, as you received them in the decision letters for each draft of your manuscript. If you agreed to publish this report and have changed your mind, or are not sure if you selected this option, please contact the editorial office as soon as possible before signing the license agreement from our publisher.

-- Reviewer comments:
Reviewer #1 (Comments for the Author):

The authors have responded satisfactorily to my comments, made appropriate changes, and added useful data to the manuscript. I have no further comments on this interesting work.
